# A molecular glue RBM39-degrader induces synthetic lethality in cancer cells with homologous recombination repair deficiency

**DOI:** 10.1038/s41698-024-00610-0

**Published:** 2024-05-24

**Authors:** Shinji Kohsaka, Shigehiro Yagishita, Yukina Shirai, Yusuke Matsuno, Toshihide Ueno, Shinya Kojima, Hiroshi Ikeuchi, Masachika Ikegami, Rina Kitada, Ken-ichi Yoshioka, Kohta Toshimitsu, Kimiyo Tabata, Akira Yokoi, Toshihiko Doi, Noboru Yamamoto, Takashi Owa, Akinobu Hamada, Hiroyuki Mano

**Affiliations:** 1 Division of Cellular Signaling, National Cancer Center Research Institute, 5-1-1 Tsukiji, Chuo-ku, Tokyo, 104-0045 Japan; 2 Division of Molecular Pharmacology, National Cancer Center Research Institute, 5-1-1 Tsukiji, Chuo-ku, Tokyo, 104-0045 Japan; 3 Department of Respiratory Medicine, Juntendo University, Graduate School of Medicine, 2-1-1 Hongo,Bunkyo-Ku, Tokyo, 113-8431 Japan; 4 Laboratory of Genome Stability Maintenance, National Cancer Center Research Institute, Tsukiji, Chuo-ku, Tokyo, 104-0045 Japan; 5 Department of General Thoracic Surgery, Juntendo University School of Medicine, 2-1-1 Hongo, Bunkyo-Ku, Tokyo, 113-8431 Japan; 6 Eisai Co., Ltd, 5-1-3 Tokodai, Tsukuba-shi, Ibaraki, 300-2635 Japan; 7 Department of Experimental Therapeutics, National Cancer Center Hospital East, Kashiwa Chiba, 277-8577 Japan; 8 Department of Experimental Therapeutics, National Cancer Center Hospital, Chuo-ku Tokyo, 104-0045 Japan; 9 Eisai Inc., 200 Metro Blvd., Nutley, NJ 07110 USA

**Keywords:** Cancer genomics, Targeted therapies, Cancer models, Targeted therapies

## Abstract

E7820 and Indisulam (E7070) are sulfonamide molecular glues that modulate RNA splicing by degrading the splicing factor RBM39 via ternary complex formation with the E3 ligase adaptor DCAF15. To identify biomarkers of the antitumor efficacy of E7820, we treated patient-derived xenograft (PDX) mouse models established from 42 patients with solid tumors. The overall response rate was 38.1% (16 PDXs), and tumor regression was observed across various tumor types. Exome sequencing of the PDX genome revealed that loss-of-function mutations in genes of the homologous recombination repair (HRR) system, such as *ATM*, were significantly enriched in tumors that responded to E7820 (*p* = 4.5 × 10^3^). Interestingly, E7820-mediated double-strand breaks in DNA were increased in tumors with BRCA2 dysfunction, and knockdown of *BRCA1/2* transcripts or knockout of *ATM*, *ATR*, or *BAP1* sensitized cancer cells to E7820. Transcriptomic analyses revealed that E7820 treatment resulted in the intron retention of mRNAs and decreased transcription, especially for HRR genes. This induced HRR malfunction probably leads to the synthetic lethality of tumor cells with homologous recombination deficiency (HRD). Furthermore, E7820, in combination with olaparib, exerted a synergistic effect, and E7820 was even effective in an olaparib-resistant cell line. In conclusion, HRD is a promising predictive biomarker of E7820 efficacy and has a high potential to improve the prognosis of patients with HRD-positive cancers.

## Introduction

Indisulam (E7070) and E7820 belong to a unique class of anticancer agents known as sulfonamide molecular glues, which selectively degrade the splicing factor RBM39. Indisulam was originally identified as an inhibitor of G1 cell cycle progression by phenotypic screening^1^, whereas E7820 was discovered as a tumor angiogenesis inhibitor^2^. These sulfonamide molecular glues have been examined in multiple phase I and II clinical trials for the treatment of solid tumors. Although these compounds showed an acceptable safety profile, only modest clinical efficacy has been observed in trials^3^, likely because neither the mechanism of action nor appropriate biomarkers for stratification have been elucidated.

Recently, these sulfonamides were revealed to act as molecular glues that induce protein complex assembly between RBM39 and DCAF15, an adapter protein for the CUL4/DDB1 E3 ubiquitin ligase^4,5^. This assembly results in the selective ubiquitination and degradation of RBM39. Based on this unique drug mechanism, new therapeutic approaches involving sulfonamides have been proposed, including achieving selective antitumor activity in leukemic cells by sharpshooting splicing factors with mutations^6^, targeting the KRAS pathway by regulating the alternative splicing of the KRAS4A isoform^7^, and inducing immunogenic peptides to elicit antitumor immunity^8^.

The patient-derived xenograft (PDX) model is a tumor-bearing mouse model created by transplanting patient tumor specimens directly into immunodeficient mice. The PDX model has attracted great attention because it accurately maintains the heterogeneity and structure of the original tumors, and the response to drug treatments in PDXs often reflects the patient’s response to the treatments^9–11^. Compared to cell lines or cell line-xenograft models, PDXs better reflect the therapeutic effect, with more than 80% similarity between clinical data and PDX responses in some instances^12^.

We have been constructing a comprehensive PDX library from Japanese patients with cancer (J-PDX library, *n* = 624 as of June 28, 2023) and have systematically used the PDX library for drug evaluation^13^. These PDX models have also been used for “coclinical studies” comparing the efficacy of the same treatments for patients and paired PDXs^14^. Evaluating drug efficacy in such PDXs will accelerate effective personalized and precise treatments. Indeed, trastuzumab deruxtecan has been shown to induce an identical response in patient tumors and matched PDXs in uterine carcinosarcoma^15,16^.

A preclinical study was performed to determine the clinical potential of E7820 using J-PDX models established from 52 patients with solid tumors. Comprehensive molecular profiling with whole-exome sequencing (WES) and RNA sequencing (RNA-seq) of such PDX tumors was further used to examine the predictive biomarkers of E7820, revealing that homologous recombination deficiency (HRD) is a novel and promising biomarker.

## Results

### PDX models for evaluating the drug efficacy of E7820

**Fig. 1:**
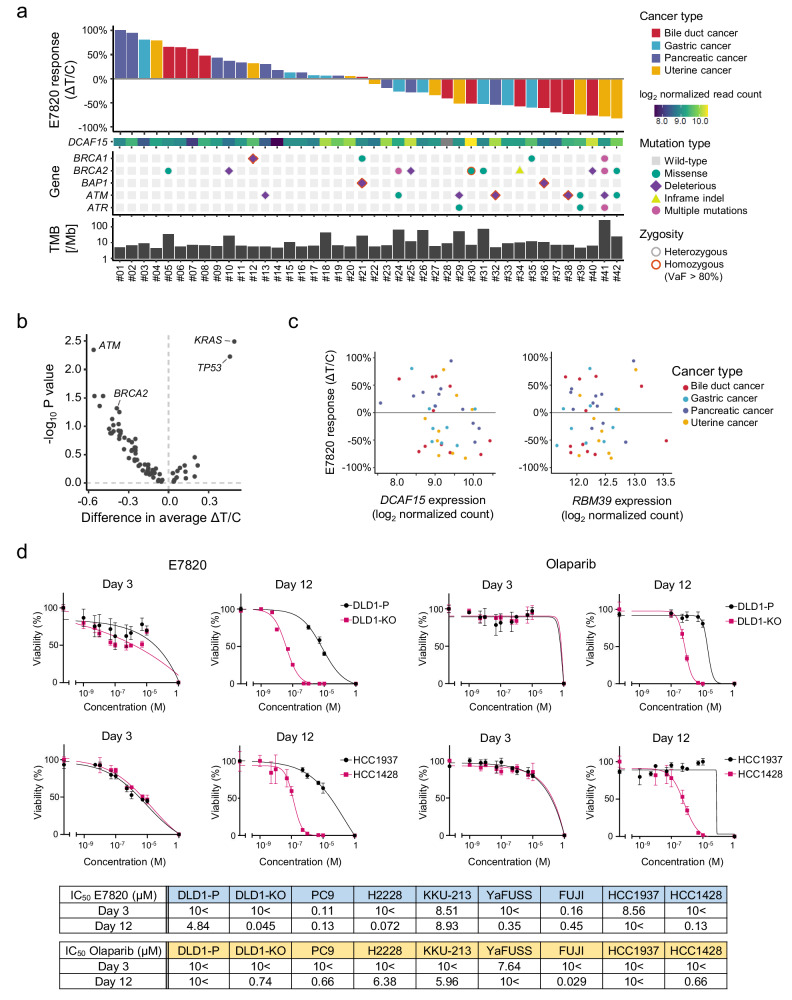
A large-scale trial using the PDX model and comprehensive molecular profiling. **a** E7820 sensitivity, HRR gene alterations, and tumor mutation burden (TMB) of 42 PDXs. **b** Volcano plot representation of Wilcoxon’s rank sum test comparing the E7820 sensitivity of PDXs with and without mutations in 73 cancer-related genes that were mutated in at least five PDXs. **c** Scatter plots showing the E7820 response and gene expression. **d** The drug efficacy in the short term (3 days) or long term (12 days) was investigated. The viability of DLD1-P, DLD1-KO, HCC1937, and HCC1428 cells was evaluated after 3 days or 12 days of treatment with E7820 or olaparib. The IC_50_ values of E7820 and olaparib in nine cell lines on day 3 or day 12 are shown in tables.

E7820 was orally administered to 42 PDX models of various types of tumors, including tumors of the bile duct (12), pancreas (12), stomach (9), and uterus (9). Significant tumor shrinkage (ΔT/C < −30%) was observed among the tumors (Fig. 1a). The overall response rates were 38.1% (16 PDX) and 54.8% (23 PDX) for 100 and 200 mg/kg of the drug administered, respectively (Supplemental Fig. 1 and Supplementary Data 1). The highest response rate for 100 mg/kg was observed in bile duct cancer (7/12, 58.3%), followed by uterine cancer (5/9, 55.6%) and gastric cancer (3/9, 33.3%). The lowest response rate was 8.3% (1/12) in pancreatic cancer. Pancreatic cancer was significantly more resistant to treatment than other tumor subtypes (HR = 11.0, 95% confidence interval (CI) = 1.26–96.2, *p* = 1.5 × 10^−2^, Fisher’s exact test).

### HRD is highly observed in E7820-sensitive PDXs

WES and RNA-seq were conducted to identify the molecular markers related to E7820 sensitivity (Supplementary Data 2 and 3). Wilcoxon’s rank sum test comparing the E7820 sensitivity of PDXs with and without mutations revealed that somatic *ATM* mutations were significantly enriched in responders (*p* = 4.5 × 10^−3^, FDR = 0.14) (Fig. 1b). *BRCA2* mutations (*p* = 4.8 × 10^−2^, FDR = 0.51), as well as mutations in *PTEN* (*p* = 2.9 × 10^−2^, FDR = 0.43), *ZNF521* (*p* = 2.9 × 10^−2^, FDR = 0.43), and *NRG1* (*p* = 4.4 × 10^−2^, FDR = 0.51) were mildly enriched in the responder group. In contrast, *TP53* was significantly mutated in non-responders (*p* = 5.9 × 10^−3^, FDR = 0.14), although this enrichment may be related to its high positivity in pancreatic cancer. Mutations in genes related to homologous recombination repair (HRR), such as *BRCA1*, *ATR*, and *BAP1*, were observed, although the number of mutant cases was insufficient for statistical analysis (Fig. 1b).

Similarly, the transcriptome was analyzed to identify the gene expression markers related to E7820 sensitivity, revealing that 1558 genes showed significantly (*p* < 0.01) decreased or increased expression in responders. In contrast to previous reports^17^, *DCAF15* was only weakly correlated with the response (*p* = 3.1 × 10^−2^, Spearman’s correlation = −0.34) (Fig. 1c).

### Cell growth inhibition becomes apparent under long-term treatment with E7820

A recent report by Xu Y. et al. indicated that E7820 affects the RNA splicing of some HRR genes but did not demonstrate that HRD can serve as a biomarker for predicting E7820 response^18^. Since our initial screening in the PDX models showed marked shrinkage of the tumors with HRD, we hypothesized that growth suppression may become dominant after long-term exposure to the compound. Drug efficacy was thus evaluated in short-term (3 days) or long-term (12 days) cultures of cancer cell lines, including a colorectal cancer cell line with (DLD1-KO) or without *BRCA2* knockout (DLD1-P). As shown in Fig. 1d, DLD1-KO cells, but not DLD1-P cells, were highly sensitive to E7820 and poly-ADP ribose polymerase inhibitor (PARPi) olaparib on day 12. Additional cancer cell lines were tested, including breast cancer cell lines with *BRCA1* mutations (HCC1428 and HCC1937) (Supplemental Fig. 2). Both compounds suppressed the growth of HCC1428 cells but not of HCC1937 cells on day 12. In a previous study, HCC1937 was reported to be resistant to olaparib (IC_50_ > 10 μM)^19,20^, suggesting that HCC1937 may have recovered from HRD status. Among other cell lines, a strong response to E7820 was observed in PC9 (lung adenocarcinoma) and FUJI (synovial sarcoma) cell lines on day 3.

### BRCA2 dysfunction increases E7820-induced DNA double-strand breaks

**Fig. 2:**
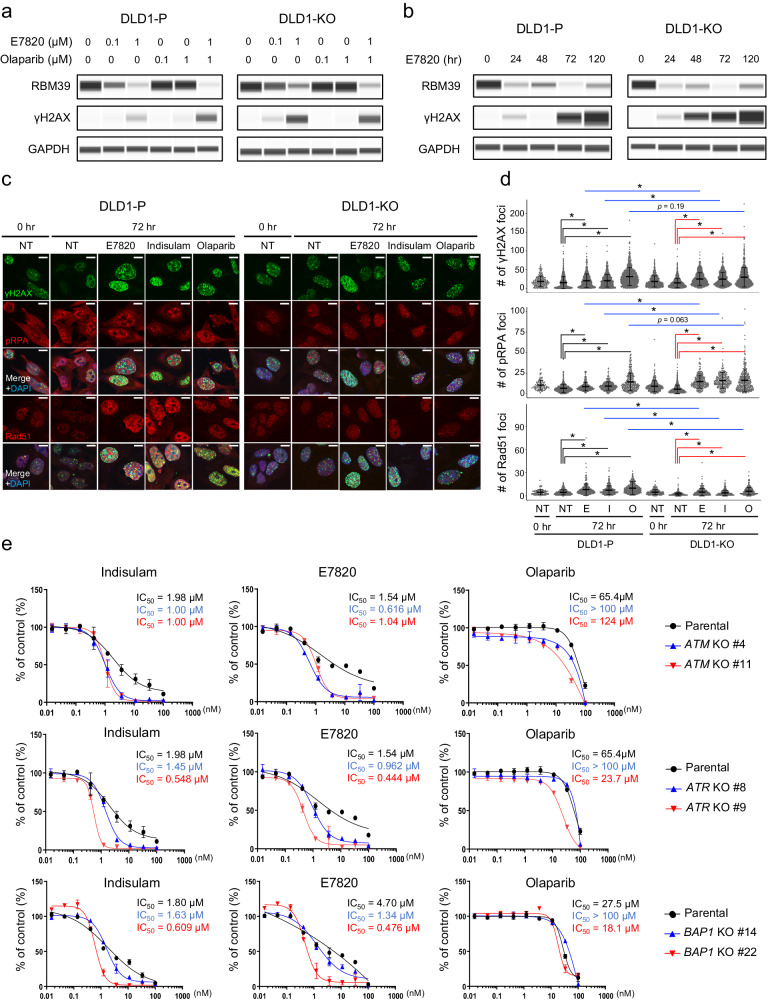
BRCA2 dysfunction increases E7820-induced DNA double-strand breaks and sensitizes cells to E7820. **a** γH2AX, a marker of DNA double-strand breaks, was evaluated by western blotting of cells treated with E7820 and olaparib. Treatment with 1 μM E7820 for 72 h degraded RBM39 protein and induced the appearance of more γH2AH than treatment with 1 μM olaparib. GAPDH was used as a loading control. **b** γH2AX induction in DLD1-P or DLD1-KO cells was evaluated by western blotting after 1 μM E7820 treatment. γH2AX induction first appeared 24 h after E7820 treatment and increased up to 120 h. GAPDH was used as a loading control. **c** DLD1 cells (P or KO) were treated with olaparib (10 µM), E7820 (1 µM), or indisulam (1 µM) for 72 h, and γH2AX, pRPA, and Rad51 foci were analyzed. Representative images are provided. NT no treatment; Scale bars in images, 10 µm. **d** The numbers of foci for γH2AX, pRPA, and Rad51 were counted and statistically analyzed using Two-tailed Welch’s *t*-test. Bars in the graph show means ± standard deviations. NT no treatment, E E7820, I indisulam, O olaparib. **e** Drug sensitivities to indisulam, E7820, and olaparib were investigated in DLD1-P and artificially generated DLD1 cell lines, in which genes were genetically engineered with a knockout (KO) of *ATM*, *ATR*, and *BAP1* by the CRISPR‒Cas9 system. Sensitivity was assessed on day 12 after drug treatment.

Since DLD1-KO showed sensitivity to E7820, similar to olaparib, exposure to E7820 may induce DNA damage. The presence of γH2AX, a marker of DNA double-strand breaks, was evaluated in cells cultured with E7820 or olaparib for 72 h. As shown in Fig. 2a, treatment with E7820 (1 µM) degraded RBM39 and induced the appearance of more γH2AX than treatment with olaparib (1 µM) (Fig. 2a). γH2AX was detectable 24 h after E7820 treatment and was markedly induced up to 120 h in DLD-KO cells (Fig. 2b). Consistent with the drug sensitivity results, γH2AX was more highly induced in DLD1-KO cells than in their parental cell lines treated with E7820 or olaparib.

The intranuclear foci of HRR-related proteins and γH2AX were further quantified in cells treated with these compounds. The number of γH2AX foci was higher in DLD1-KO cells than in DLD1-P cells, and this difference was further enhanced by treatment with olaparib, E7820, or indisulam (Fig. 2c). A similar trend was observed for foci of phosphorylated RPA (pRPA) (Fig. 2c and d). In contrast, RAD51 foci were decreased in DLD1-KO cells compared to those in DLD1-P cells. These observations indicate that DNA double-strand breaks are retained in DLD1-KO cells because RAD51 filaments are not successfully formed without functional BRCA2.

KKU-213, an HR-proficient cholangiocarcinoma cell line, was used to examine whether suppression of *BRCA1* or *BRCA2* expression sensitizes the cells to E7820. Three different shRNAs were tagged with an enhanced green fluorescent protein (EGFP) and placed under a doxycycline-inducible promoter. As shown in Supplemental Fig. 3, the mRNA levels of *BRCA1* or *BRCA2* dropped to 20–40% with corresponding shRNA induction compared to that in cells with the control shRNA. Interestingly, the fraction of EGFP-positive cells gradually decreased in a time-dependent manner when the cells were incubated with E7820 or olaparib, again confirming BRCA1/BRCA2-dependent E7820 sensitivity (Supplemental Fig. 3). Notably, shRNA-targeting luciferase did not affect the EGFP-positive cell fraction.

To further examine whether other genes involved in the DNA damage response affect sensitivity to E7820, *ATM*, *ATR*, or *BAP1* genes were knocked out in DLD1 cells, which were then subjected to drug exposure. As shown in Fig. 2e, the deletion of *ATM*, *ATR*, or *BAP1* sensitized DLD1 cells to E7820 and indisulam, while changes in sensitivity to olaparib were not clear.

### Transcriptomic changes associated with E7820

**Fig. 3:**
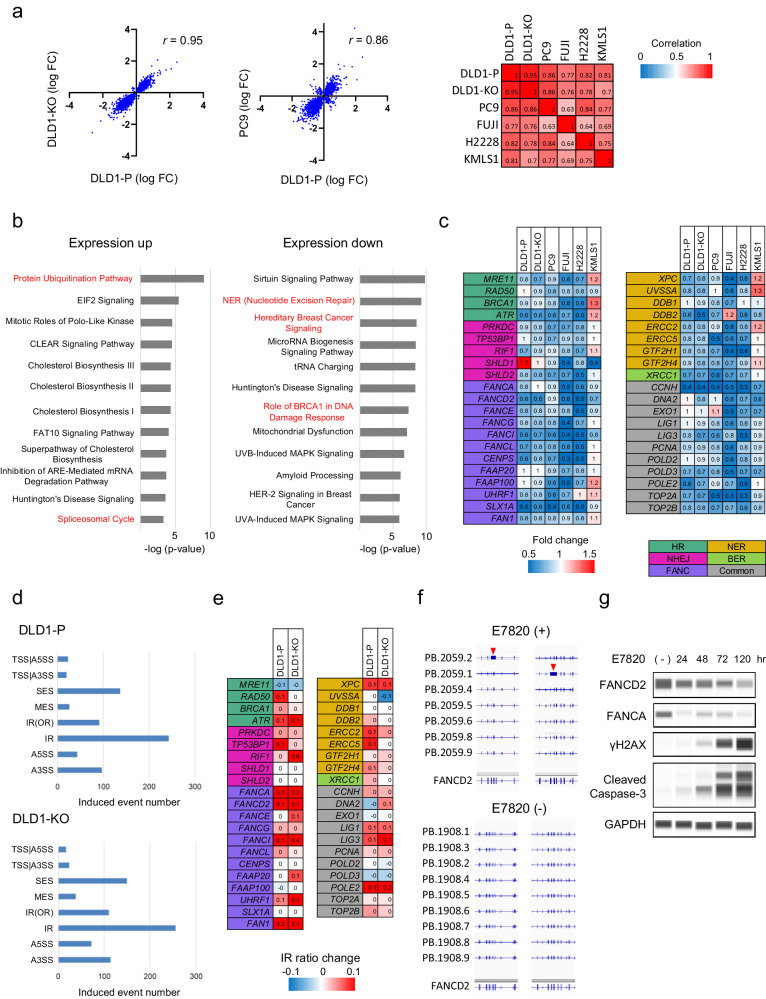
Transcriptomic changes associated with E7820. **a** Transcriptomic changes associated with E7820. RNA-seq was performed in six cell lines to compare the changes in gene expression induced by E7820. The expression changes (log fold change; log FC) were highly concordant between DLD1-P and DLD1-KO cells (*r* = 0.95, left panel) or PC9 cells (*r* = 0.86, middle panel). The heatmap on the right shows that the trends in expression profile changes associated with E7820 were similar between any pair of DLD1-P, DLD1-KO, PC9, FUJI, H2228, and KMLS1. **b** The genes that were commonly upregulated (fold change > 1.1) and downregulated (fold change < 0.9) by E7820 treatment among the six cell lines included 1655 and 2787 genes, respectively. Ingenuity pathway analysis (IPA) was conducted for pathway analysis. Among the identified pathways shared by upregulated genes, “Protein Ubiquitination Pathway” and “Spliceosomal Cycles” were highly ranked (−log(*p* value) = 9.01 and 3.33, respectively). In contrast, the enriched pathways of the downregulated genes included pathways related to DNA damage repair, such as “Nucleotide excision repair (NER) Enhanced Pathway”, “Hereditary Breast Cancer Signaling”, and “Role of BRCA1 in DNA Damage Response” (−log(*p* value) = 9.27, 8.51 and 7.38, respectively). **c** The changes in gene expression induced by E7820 treatment are shown. The fold changes are shown in a heatmap comparing the expression of cell lines after E7820 treatment (1 μM for 48 h) to basal expression. The genes are involved in homologous recombination repair (HRR), nonhomologous end joining (NHEJ), the Fanconi anemia complementation group (FANC) pathway, nucleotide excision repair (NER), base excision repair (BER) and other pathways related to DNA damage repair (Common). Only genes whose expression was decreased by 10%< are shown. Information on all genes is shown in Supplemental Fig. 4. **d** The number of splicing anomalies induced by E7820 treatment is indicated. Similar alternative splicing events were induced by E7820 in both DLD1-P and DLD1-KO cells. Mis-splicing event codes are as follows: TSS transcription start site, A5SS alternative 5’ splice site, A3SS alternative 3’ splice site, SES single-exon skipping, MES multiple-exon skipping, IR intron retention, OR overlapping region. **e** The changes in IR ratio induced by E7820 treatment are shown. The most significant changes in individual genes of Fig. 3c are shown in a heatmap comparing the IR ratios of cell lines after E7820 treatment (1 μM for 48 h) to basal ratio. **f** Full-length cDNAs were selectively prepared from the cells and sequenced with a Sequel II long-read sequencer (Pacific Biosystems). The long-read sequences were used to identify isoforms using the bulk Iso-seq workflow and pigeon workflow. The isoforms were visualized using IGV (Integrative Genomics Viewer). The individual long reads were tagged with PB ID, as shown on the left. Intron retention within *FANCD2* was specifically observed in the cells treated with E7820 (arrowheads). **g** The protein levels of FANCD2 and FANCA in DLD1-KO cells. DLD1-KO cells were incubated with E7820 for the indicated times, and western blotting was conducted to evaluate the protein expression of FANCD2, FANCA, γH2AX, and cleaved caspase-3.

The RNA-seq dataset was further evaluated to clarify how E7820 induces DNA double-strand breaks. First, the changes in gene expression induced by E7820 were highly concordant between DLD1-P and DLD1-KO cells (*r* = 0.95) (Fig. 3a). Similarly, the trends in expression profile changes associated with E7820 were similar between DLD1-P, DLD1-KO, PC9, FUJI, H2228, and KMLS1 (Fig. 3a and Supplementary Data 4). These results suggest that E7820 causes similar changes in gene expression irrespective of the genomic background of the cells.

We then isolated genes commonly upregulated or downregulated by E7820 treatment among the six cell lines. There were 1655 upregulated genes (fold change > 1.1) and 2787 downregulated genes (fold change < 0.9) observed in more than three cell lines, as annotated via ingenuity pathway analysis (IPA). Among the identified pathways shared among the upregulated genes, “Protein Ubiquitination Pathway” and “Spliceosomal Cycles” were highly ranked (−log(*p*-value) = 9.01 and 3.33, respectively). These results are reasonable, considering that the mode of action of E7820 is related to the ubiquitination of RBM39, an RNA splicing factor. In contrast, the enriched pathways of the downregulated genes included those related to DNA damage repair, such as “Nucleotide excision repair (NER) Enhanced Pathway,” “Hereditary Breast Cancer Signaling,” and “Role of BRCA1 in DNA Damage Response” (−log(*p* value) = 9.27, 8.51 and 7.38, respectively) (Fig. 3b). These genes included HRR-related genes, such as *PALB2*, *BRIP1*, *BRCA1*, *RAD50*, *MRE11*, *ATR* and *FANC* family genes (Fig. 3c and Supplemental Fig. 4). Furthermore, downregulated genes also included essential genes for NER, such as *XPC* and *ERCC2*.

Next, we investigated the alternative splicing events induced by E7820. In both DLD1-P and DLD1-KO, intron retention was the most increased splicing anomaly induced by E7820, while the most decreased splicing anomaly was single-exon skipping (Fig. 3d and Supplemental Fig. 5a). The most common total (induced and reduced) anomaly was exon skipping by both PSI-sigma and rMATS (Supplemental Fig. 5b). This result was concordant with the previous study^21^.

The intron-retained genes thus identified were also subjected to IPA, revealing that the shared pathways among them included “Hereditary Breast Cancer Signaling” and “Role of BRCA1 in DNA Damage Response” (Supplemental Fig. 6). To confirm the characteristics of the transcripts in E7820-treated cells, full-length cDNAs were selectively prepared from cells and sequenced using a Sequel II long-read sequencer (Pacific Biosystems, Menlo Park, CA, USA). Among 41 genes related to DNA repair shown in Fig. 3c, intron retention was induced in 25 genes (61%) in DLD1-KO cells (Fig. 3e and Supplemental Fig. 7). Among them, novel intron retentions within *FANCD2*, *BRCA1*, *FANCA*, *ERCC5*, *FAN1*, *FANCG*, *FANCI*, *XPC* were observed in cells treated with E7820 (Fig. 3f and Supplemental Fig. 7).

Any missplicing of mRNAs severely affects the translation of the corresponding mature proteins. Therefore, we measured the protein levels of FANCD2 and FANCA in DLD1-KO cells (Fig. 3g). Incubation with E7820 markedly reduced the levels of FANCD2 and FANCA proteins in a time-dependent manner (although the rate of suppression varied). Importantly, a decrease in FANCD2/FANCA protein levels coincided with the accumulation of γH2AX and cleavage of caspase-3 (a hallmark of cell apoptosis).

We further knocked down *FANCA* and *FANCD2* in DLD1-P cells to investigate if their depletions sensitize tumor cells to E7820. The IC_50_ to E7820 was not significantly changed in the cells with shRNA of *FANCA* and *FANCD2* (Supplemental Fig. 8). As intron retention was induced in many genes related to DNA damage repair, not only a few of the genes could lead the cells sensitive to E7820.

### Combination treatment with E7820 and olaparib or cisplatin

**Fig. 4:**
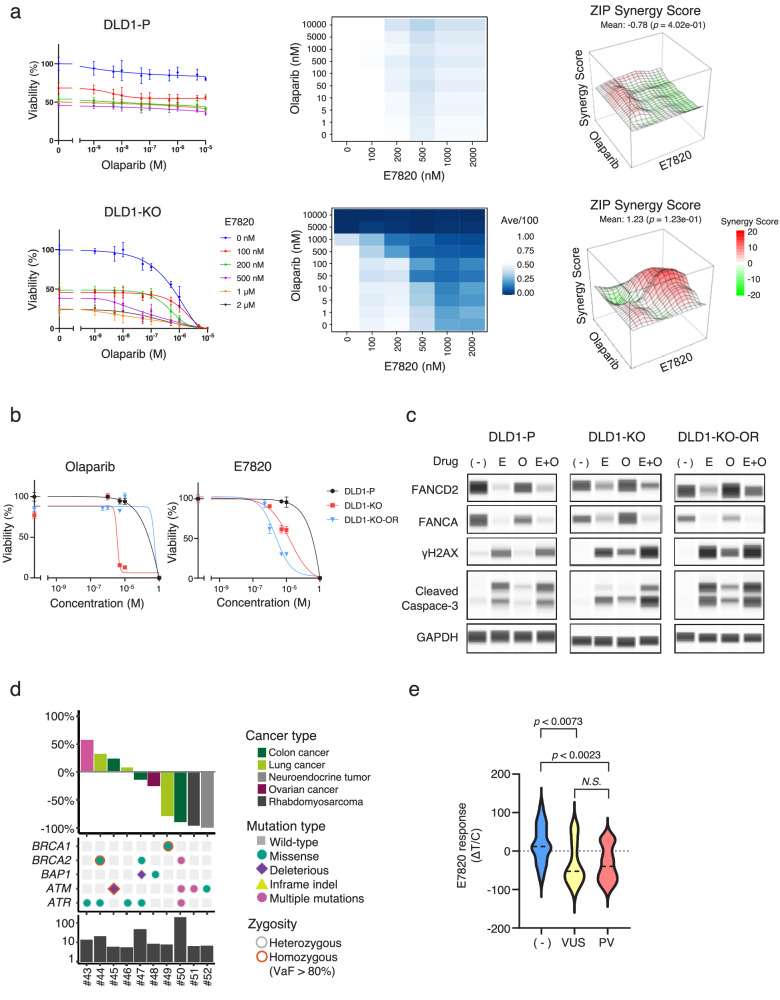
Combination treatment with E7820 and olaparib or cisplatin. **a** Combination therapy with E7820 and olaparib. DLD1-P and DLD1-KO cells were treated with a combination of E7820 and olaparib at the indicated concentrations. Cell viability was assessed with a PrestoBlue cell viability assay. Synergistic effects are indicated in the 3D drug synergy maps. The surfaces of the maps were color-coded according to the ZIP synergy scores of the combination treatment. **b** The olaparib-resistant DLD1-KO cell line (DLD1-KO-OR) was developed through treatment with gradually increasing concentrations of olaparib (upto 20 μM). The resistant cell was treated with E7820 or olaparib for 12 days, and cell viability was assessed. **c** DLD1-P, DLD1-KO, and DLD1-KO-OR cells were treated with E7820 (1 μM), olaparib (1 μM), and their combination. The protein expression of the indicated genes was evaluated by western blotting. **d** PDXs with any HRR gene mutations were treated with E7820 (100 mg/kg). E7820 response (ΔT/C), HRR gene alterations, and TMB of 10 PDXs are shown. **e** A total of 52 PDX models were divided into three groups, and E7820 response (ΔT/C) were compared. (−) no mutations, VUS variant of unknown significance, PV pathogenic variants in HRR genes.

Compared to olaparib, a specific inhibitor of PARP1/2, E7820 appears to inhibit a broad range of genes related to DNA damage repair. To evaluate whether a combination treatment with E7820 and olaparib or cisplatin has an additional effect, a drug sensitivity assay was conducted in DLD1-P or DLD1-KO cells. E7820 and olaparib/cisplatin exerted a mild synergistic killing effect only on DLD1-KO cells at concentrations around the IC_50_ (500 nM of E7820 and 50 nM of olaparib) and not on DLD1-P cells (Fig. 4a, Supplemental Fig. 9, Supplemental Fig. 10 and Supplementary Data 5).

We then developed an olaparib-resistant DLD1-KO cell line (DLD1-KO-OR) by treatment with gradually increasing concentrations of olaparib. Interestingly, E7820 was still capable of inducing cell death in DLD1-KO-OR cells, suggesting that the mechanism of resistance to olaparib may not affect E7820 sensitivity (Fig. 4b). DLD1-P, DLD1-KO, and DLD1-KO-OR cells were treated with E7820 (1 μM), olaparib (1 μM), or a combination of both. FANCA and FANCD2 protein levels were depleted following treatment with E7820. Subsequent increases in γH2AX levels and caspase-3 cleavage were observed in all cells (Fig. 4c).

To investigate whether HRR deficiency induced by E7820 might attenuate repair competence, HRR was evaluated. DLD1 cells (P or KO) were treated with E7820 (1 µM) or indisulam (1 µM) during and after hydroxyurea (2 mM) treatment, which induced double-strand breaks, and γH2AX and pRPA foci were analyzed (Supplemental Fig. 11). The number of γH2AX and pRPA foci in E7820-treated tumors was greater than that in the untreated group (Supplemental Fig. 11).

### Proof of concept in a validation PDX cohort

Because HRD is a promising biomarker for E7820 treatment, we further evaluated E7820 efficacy in additional PDX models involving any mutation within *BRCA1*, *BRCA2*, and *ATM* as a validation cohort. E7820 was administered orally to 10 PDXs of various types of tumors, including colon, lung, ovarian, sarcoma, and neuroendocrine tumors. Significant tumor shrinkage (ΔT/C < −30%) was observed in four of these tumors (Fig. 4d and Supplemental Fig. 12). Responsive tumors included colon cancer, lung cancer, sarcoma, and neuroendocrine tumors. Finally, a total of 52 PDX models were divided into three groups: PDXs with pathogenic variants, variants of unknown significance (VUS), and no mutations in HRR genes. PDXs with pathogenic variants in HRR genes were significantly more sensitive to E7820 (100 mg/kg) than those with no mutations in HRR genes (*p* < 0.001) (Fig. 4e).

## Discussion

We utilized our J-PDX library to search for predictive biomarkers for the molecular glue RBM39 degrader E7820. By integrating the drug response rate into the WES and RNA-seq datasets, we unexpectedly revealed that HRD may be useful in selecting sensitive tumors for E7820 treatment. Previous studies have reported that the expression of *DCAF15*, a substrate adaptor in the E3 ligase complex for RBM39 degradation, serves as a biomarker for E7820^17,22^. While *DCAF15* is one of the essential components required for the drug to function, our transcriptome data indicated only a weak correlation (*p* = 0.03) between the drug response and *DCAF15* expression. Therefore, *DCAF15* expression alone may not be sufficient to stratify cancer patients for E7820 treatment.

Although it is difficult to accurately annotate the pathogenicity of VUS in HRR genes, PDXs with apparent pathogenic variants are more sensitive to E7820 than non-mutant PDXs. “Two hits” are known to be necessary for tumor suppressor genes to indicate a phenotype^23^. However, the heterogeneous loss of HRR genes (haploinsufficiency) could be a potential target of E7820 as it could globally decrease the expression of DNA repair genes.

In a previous phase 1 trial of E7820, 37 patients with solid tumors were enrolled, and 29 patients were evaluated for response. Although no partial or complete responses were observed, eight patients showed stable disease (≥4 months), including five patients with protracted stable disease exceeding 6 months^24^. Notably, the study did not selectively recruit patients with HRD-positive tumors or specific tumor types.

In acute myeloid leukemia (AML), it has been reported that the degradation of RBM39 represses cassette exon inclusion and promotes intron retention within mRNAs encoding HOXA9 targets and in other RNA-binding proteins (RBPs) preferentially required for AML survival^6^. However, the splicing targets of RBM39 in solid tumors are largely unknown. We discovered that intron retention was selectively observed in genes within the DNA damage repair pathways, although the precise mechanism by which RBM39 regulates these genes remains to be elucidated. A previous study has indicated that the small pyrimidine stretch (SPY) represents a potential binding site for the U2AF large subunit and RBM39 tandem RNA recognition motifs (RRM)^25^.

A recent study demonstrated that the loss of RBM39 induces splicing defects in key DNA damage repair genes, such as *ATM* and *BRCA1,* in ovarian cancer, leading to increased sensitivity to cisplatin and various PARPi^18^. Although this study is similar to ours, a few important contexts are different. First, our study identified that HRD could be a promising predictive biomarker of E7820, while the authors of the other group did not consider BRCA and HRD status as predictive markers for RBM39-targeting molecular glues. By evaluating the long-term treatment effects, we noticed a significant effect of HRD status on E7820 efficacy. Second, as the predictive biomarker for E7820 was identified in our study, we expect that this compound is not only useful for ovarian cancer in combination with PARPi but also potentially applicable to various tumor types and indications.

The clinical implications of our results may be related to the following four applications, although further clinical investigation should be performed for validation. First, tumors with HRD should be appropriate targets for E7820. Compared to PARPi, E7820 targets multiple genes in DNA damage repair pathways. Indeed, tumors with mutations in *ATM*, *ATR*, or *BAP1,* in addition to *BRCA1/2*, were effectively killed with E7820. Second, a novel combination therapy involving E7820 and PARPi is warranted, although the potential adverse events induced by PARPi should be carefully managed, including hematotoxicity and interstitial pneumonia^26–28^. Because the target genes of both drugs overlap but also differ, combination treatments may have synergistic effects with individual drugs, as shown in our in vitro assessment (Fig. 4a). Third, E7820 can be used as a drug for treating olaparib-refractory cancers. Several mechanisms of PARPi resistance have been identified, including the restoration of HRR proficiency, switching to alternate repair mechanisms, such as nonhomologous end joining (NHEJ), replication fork stabilization, drug efflux, and decreased PARP expression^29–31^. It remains unclear which resistance mechanisms are sensitive to E7820. Although our olaparib-resistant cell line retained sensitivity to E7820, HRR proficiency due to reversion mutations in *BRCA1/2* may lead to E7820 resistance. Finally, E7820 could be applied to the treatment of cholangiocarcinoma, for which limited molecular-targeted therapies, such as FGFR inhibitors, are currently available^32,33^. E7820 was preferentially effective against this tumor subtype.

This study had several limitations. First, it was not confirmed whether RBM39 directly regulated RNA splicing among the genes considered in this study. However, E7820 treatment induced similar changes in gene expression irrespective of the cell line. Therefore, E7820 likely targets a master regulator of splicing of a specific gene set. Second, not all predictive biomarkers for E7820 have been identified. In particular, other mechanisms may also underlie the rapid induction of apoptosis observed in some cell lines, such as PC-9 and FUJI. Third, the PDX models suggested that pancreatic cancer may be resistant to E7820; however, the underlying mechanism is not clear. Genetic and epigenetic factors may also regulate apoptosis. Fourth, additional validation is needed to investigate if pathogenic mutations in *ATM*, *ATR*, and *BAP1* are the predictive markers for E7820, as only one cell line was used in this study. Finally, although a mild synergistic effect was observed in the combinational treatment of E7820 and olaparib, in vivo assessment remains to be investigated.

In conclusion, we discovered novel predictive biomarkers for E7820, which should facilitate further development of E7820 applications. Our PDX library assessment highlighted the importance of a well-designed model system, and it is essential to identify potential biomarkers for clinical trial design. We hope that this study paves the way for the application of this novel class of anticancer drug, RBM39 degraders, in patients with HRD-positive cancer and to improve their prognosis.

## Methods

### Study design

In this study, we used PDX models stored in the J-PDX library, which we have previously reported^13^. All experiments were performed according to a protocol approved by the National Cancer Center Institutional Review Board (2015-123), and precepts were established by the Helsinki Declaration. The study design and conduct complied with all applicable regulations, guidance, and local policies. Written informed consent was obtained from all human participants. Samples of participants who opted out to participate were not used in this study. Animal experiments were performed in compliance with the guidelines of the Institute for Laboratory Animal Research, National Cancer Center Research Institute (T17-073 and T19-008).

### Animal study

Frozen tumor samples obtained from the J-PDX library were implanted subcutaneously in the flanks of 6-week-old female NOG mice (NOD. Cg-Prkdcscid Il2rgtm1Sug/ShiJic, In-Vivo Science Inc., Kanagawa, Japan). Once the transplanted tumors had grown, they were removed and transferred to an appropriate number of mice for further drug efficacy testing. Mice were housed in sterile, filter-capped, polycarbonate cages, maintained in a barrier facility under a 12-h light/dark cycle, and provided with sterilized food and water. All invasive procedures were performed with intraperitoneal administration of three types of mixed anesthesia (medetomidine hydrochloride, Meiji Seika Pharma, Tokyo, Japan; midazolam, Maruishi Pharmaceutical Co., Osaka, Japan; betorfal tartrate, Meiji Seika Pharma) or inhalation of isoflurane (Zoetis Japan, Tokyo, Japan) to reduce pain in the experimental animals.

### Drug efficacy test for E7820 in the PDX model

Drug efficacy tests for E7820 (provided by Eisai Co., Ltd., Ibaraki, Japan) were performed across different cancer types, including bile duct cancer, gastric cancer, pancreatic cancer, uterine cancer, colon cancer, lung cancer, neuroendocrine tumor, ovarian cancer, and sarcoma. To reduce or eliminate distress to the animals, they were euthanized by cervical dislocation after the observation period was complete, or when they showed rapid weight loss of 20% or more over several days, or when the tumor volume reached 10% or more of the body weight. The tumor volume and body weight were measured twice per week. Tumors were grouped into three groups when the tumor volume reached an average of 150–250 mm^3^. At the time of grouping, software calculations (EXSUS software, CAC Croit Corporation, Tokyo, Japan) were performed to minimize differences in tumor volume between the groups. Each treatment set included the following treatments: no treatment (control), 100 mg/kg E7820, or 200 mg/kg E7820 (dissolved in 3.5% DMSO, 6.5% Tween 80 in 5% glucose) administered orally daily. All animals were euthanized on day 21 or when the humane endpoint criterion was met, whichever occurred first. The antitumor effect was presented as the ratio of growth (ΔT/C) based on comparing the ratio of the tumor increment on a specific date to the start date (relative tumor growth, RTV) in the control and treatment groups, as follows:

If the RTV of the treatment group was >1:

ΔT/C = (average RTV of treatment group−1)/(average RTV of control group−1)

If the RTV of the treatment group was <1:

ΔT/C = (average RTV of the treatment group−1)

### Cell lines

Human colorectal adenocarcinoma cell line DLD1 parental cells and homozygous *BRCA2* (−/−) variant cells were purchased from Horizon Discovery, Inc. (Cambridge, UK). PC9 was provided by the RIKEN BRC (Ibaraki, Japan) through the National BioResource Project of the MEXT, Japan. KKU-213 and KMLS1were provided from the JCRB Cell Bank. (Osaka, Japan). YaFuSS, derived from synovial sarcoma, was kindly provided by Dr. J. Toguchida (Kyoto University, Japan). FUJI was a gift from Dr. S. Tanaka (Hokkaido University, Japan). H2228, HCC1937, and HCC1428 were purchased from the American Type Culture Collection (Manassas, VA, USA). Cells were cultured in RPMI-1640 (Fujifilm Wako Pure Chemical Corporation, Tokyo, Japan) supplemented with heat-inactivated 10% fetal bovine serum (Merck, Rahway, NJ, USA), 100 U/mL penicillin, and 100 μg/mL streptomycin (Fujifilm Wako Pure Chemical Corporation) at 37 °C and 5% CO_2_ in a CO_2_ incubator. All cell lines were used for the experiments within ten passages from thawing. Cell line authentication and Mycoplasma testing were not carried out within six months.

### shRNA knockdown and cell competition assay

For the de novo generation of miR-E shRNAs, 97-mer oligonucleotides (Eurofine, Luxembourg City, Luxembourg) encoding the respective shRNAs were PCR amplified using the primers miRE-Xho-fw (5′- TGAACTCGAGAAGGTATATTGCTGTTGACAGTGAGCG-3′) and miRE-EcoOligo-rev (5′-TCTCGAATTCTAGCCCCTTGAAGTCCGAGGCAGTAGGC-3′) and 0.05 ng oligonucleotide template and cloned into miR-E recipient vectors (LT3GEPIR, a gift from Johannes Zuber, Addgene plasmid # 12345) according to a previous study^34^ (Supplemental Fig. 3a). Cell lines were retrovirally transduced under single-copy conditions and competitive proliferation assays were performed as described previously study^35,36^. Enhanced green fluorescent protein (EGFP) expression, which uses the same promoter with shRNA, was quantified in shRNA-expressing (GFP+) cells by flow cytometry after 3–21 days and compared to in-sample GFP negative control cells and parallel samples harboring control shRNAs targeting Luc with neutral potency.

### Generation of KO cells

*ATM*, *ATR*, and *BAP1* were knocked out in DLD1 cells using the CRISPR‒Cas9 system. Guide RNAs (gRNAs) were designed using Integrated DNA Technologies (IDT; Coralville, IA, USA). The guide RNA sequences for each target are as follows: ATM: 5’-ATTTATATCCATCATCCGAA-3’, ATR: 5’-GGTTGTGTTCTGCTAGAGTA-3’, and BAP1:5’- GAAGTCCTTCATGCGACTCA-3’. Alt-R S.p. Cas9-GFP V3 (IDT #10008161) and each sgRNA were mixed to prepare the RNP complex. In vitro electroporation was performed using NEPA21 (Nepa Gene, Chiba, Japan) to introduce the RNP complex into 1.0 × 10^6^ DLD1 cells with the following settings: perforation pulse: Pp voltage 150 V, Pp pulse width 5 ms, pulse interval 50 ms × 2 times, and introduced pulse: Tp voltage 20 V, pulse width 50 ms, pulse interval 50 ms × 5 times. Thereafter, each cell line was seeded in 6-well plates. The next day, the individual cells with the highest 15% GFP signals were isolated and seeded into 96-well plates using FACS. Single cells were cultured in conditioned media for approximately 3 weeks. Loss of target protein expression was confirmed by western blot analysis. Antibodies against ATM, ATR, and BAP1 were purchased from Cell Signaling Technology (Danvers, MA, USA) (ATM, #2873; ATR, #2790; and BAP1, #13975).

### Cell viability assay

Cancer cells (except for those BAP1 KO) were seeded in 96-well plates at a density of 5.0 × 10^2^ cells/well with 100 µL of medium/well, and each drug was added at various concentrations on the next day, followed by incubation at 37 °C for 3 or 12 days. E7820 and Indisulam were provided by Eisai, and olaparib and cisplatin were purchased from Selleckchem (Houston, TX, USA). DMSO (Nacalai Tesque, Kyoto, Japan) was added to a final concentration of 0.01% (volume/volume) in the wells without drugs. BAP1 KO #14 and #22 clones were plated at a seeding density of 3.0 × 10^2^ cells/well with 100 µL of medium per well. Ten microliters of PrestoBlue cell viability reagent (Thermo Fisher Scientific, Waltham, MA, USA) or CellTiter-Glo assay reagent was added to each well after exposure to these drugs, and fluorescence intensity was measured using a 2030 ARVO X3 microplate reader and PerkinElmer 2030 Software v4.0 (PerkinElmer, Waltham, MA, USA) (excitation: 530 nm, emission: 590 nm). Wells without cells were used as negative controls, and survival data were graphically analyzed as a sigmoid curve using the GraphPad Prism software for Mac (GraphPad Software, San Diego, CA, USA).

### WES

Fresh frozen samples (500 ng) were subjected to target fragment enrichment using a Twist Library Preparation EF Kit (Twist Bioscience, South San Francisco, CA, USA). Massively parallel sequencing of the isolated fragments was performed using the paired-end option on a NovaSeq 6000 platform (Illumina, San Diego, CA, USA). Paired-end WES reads with nucleotides masked with a quality score <20 were aligned to a merged reference sequence of the human reference genome (GRCh38) and *Mus musculus* reference genome (mm10) using BWA-MEM. Somatic mutations were called using the Genome Analysis Toolkit (https://gatk.broadinstitute.org/hc/en-us), MuTect2, VarScan2 (http://varscan.sourceforge.net), and our in-house somatic caller. Mutations were discarded if any of the following criteria were met: read depth <20, variant allele frequency <0.05, mutation occurring in only one strand of the genome, mutant read number in germline control samples >2, or the variant was present in normal human genomes in either the 1000 Genomes Project dataset (https://www.internationalgenome.org/) or our in-house database. Gene mutations were annotated using SnpEff (http://snpeff.sourceforge.net).

### RNA-seq

Total RNA was extracted from freshly frozen samples using RNA-Bee (Tel-Test Inc., Gainesville, FL, USA), treated with DNase I (Thermo Fisher Scientific), and subjected to poly(A)-RNA selection prior to cDNA synthesis. The RNA-seq library was prepared using the NEBNext Ultra Directional RNA Library Prep Kit (NEB, Ipswich, MA, USA) according to the manufacturer’s protocol. Sequencing was conducted from both ends of each cluster using the HiSeq 2500 or NextSeq platforms (Illumina). RNA-seq data were aligned to the GRCh38 reference genome using STAR (2.7.10b; https://github.com/alexdobin/STAR). The expression levels of the genes corresponding to GENCODE v42 were quantified using RSEM(v1.3.1; https://github.com/deweylab/RSEM). Alternative splicing events were detected using PSI-Sigma v1.9o (https://github.com/wososa/PSI-Sigma) with the default settings. Intron retention events were identified using IRFinder v1.3.1 (https://github.com/williamritchie/IRFinder) with default settings in the FASTQ mode. Additionally, differential intron retention analysis of control and treated samples was performed using the DESeq2 R package, considering padj < 0.05 as statistically significant.

### Immunofluorescence

The cells were cultured on coverslips in 24-well plates. At the indicated time points, the cells were fixed with 4% paraformaldehyde for 10 min and washed with PBS. The fixed cells were permeabilized with 0.1% Triton X-100/PBS for 15 min on ice, incubated with blocking buffer (2% goat serum in 0.3% Triton X-100/PBS) for 10 min, and washed with PBS. The cells were incubated with the primary antibodies mouse anti-γH2AX (#613402, BioLegend, San Diego, CA, USA), rabbit anti-Rad51 (#ab133534, Abcam, Cambridge, UK), rabbit anti-phospho RPA2 (Ser33) (#NB100-544, Novus Biologicals, Littleton, CO, USA), or rabbit anti-53BP1 (#PC712, Merck) in blocking buffer for 60 min. After washing with PBS, the cells were incubated with the corresponding secondary antibodies, including goat anti-mouse IgG (Alexa Fluor 488) (#ab150113, Abcam) or goat anti-rabbit IgG (Alexa Fluor 594) (#ab150080, Abcam) in 0.3% Triton X-100/PBS for 60 min in the dark. The cells were then washed with PBS, and coverslips were mounted on slides with ProLong Diamond Antifade Mountant with DAPI (Thermo Fisher Scientific). Immunofluorescence imaging was conducted using a confocal laser microscope (Olympus, FV10i, and FV3000, Shinjuku, Tokyo, Japan).

### Western blotting

Proteins were extracted on ice using 1% NP-40 lysis buffer containing protease and phosphatase inhibitors. Protein separation and detection were performed using an automated capillary electrophoresis system (Simple Western system and Compass software; ProteinSimple). Antibodies against the following proteins were used: RBM39 (1:50, HPA001591, atlas antibodies, Bromma, Sweden), phospho-histone H2A. X (Ser139) (1:50, 20E3, #9718, Cell Signaling Technology), FANCA (1:50, D1L2Z, #14657, Cell Signaling Technology), FANCD2 (1:50, D5L5X, #16323, Cell Signaling Technology), and GAPDH (1:50, 14C10, #2118, Cell Signaling Technology). Signals were detected with an HRP-conjugated secondary anti-rabbit antibody and visualized using ProteinSimple software. The uncropped scans of the blots are indicated in Supplemental Fig. 13.

### Combinatorial synergy analysis

Synergistic or antagonistic drug effects were analyzed based on the zero interaction potency (ZIP), Bliss, the highest single agent (HSA), and Loewe model^37^. Individual scores, the excess inhibition rate over the expected effect, were calculated across the entire dose–response matrix data using the R package Synergyfinder version 3.4.5^38^.

### Reporting summary

Further information on research design is available in the Nature Research Reporting Summary linked to this article.

### Supplementary information

Supplementary Information

Supplementary Data 1

Supplementary Data 2

Supplementary Data 3

Supplementary Data 4

Supplementary Data 5

Reporting summary

## Data Availability

We deposited the raw sequencing data under accession number hum0317.v1 in the Japanese Genotype–Phenotype Archive, which is hosted by the DNA Data Bank of Japan under accession number JGAS000707.

## Code availability

No unreported custom computer codes or algorithms were used to generate the results in this manuscript. The references for all source codes are included in the “Methods” section.
