# Supplementary material for: A molecular glue RBM39-degrader induces synthetic lethality in cancer cells with homologous recombination repair deficiency
Source: NPJ Precis Oncol. 2024 May 24;8:117. doi: 10.1038/s41698-024-00610-0 (PMC11126574; doi:10.1038/s41698-024-00610-0)
Supplement: Supplementary file 1 — Supplementary Information [file 41698_2024_610_MOESM1_ESM.pdf]

Supplementary Information

A molecular glue RBM39-degrader induces synthetic lethality in cancer cells with homologous recombination repair deficiency

Contents:

Supplementary Figure 1-13

a

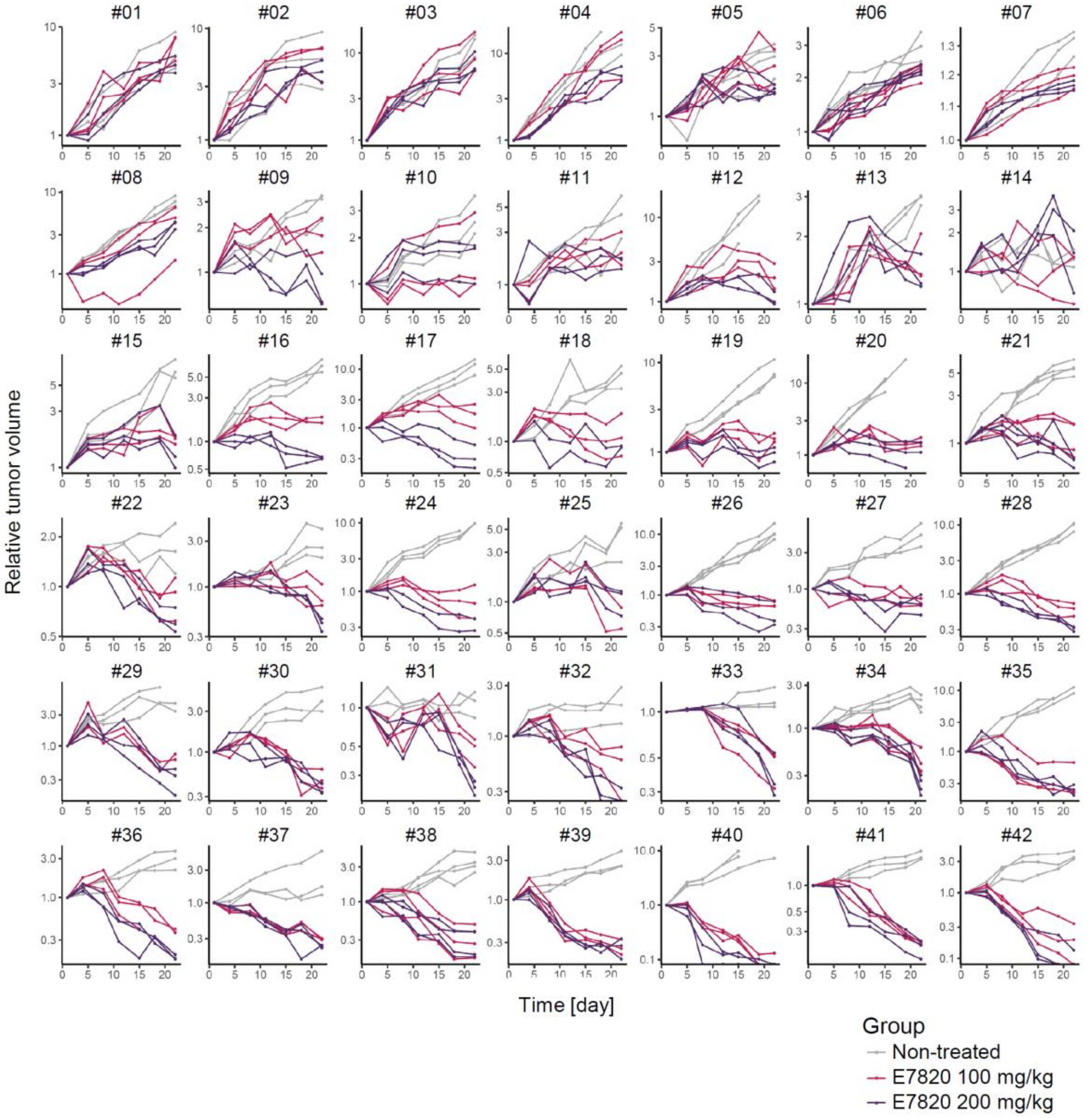

Supplemental Fig. 1. PDX models for evaluating the drug efficacy of E7820.

(a) The growth curves of E7820-treated PDXs shown in **Fig. 1a**. Each line indicates one mouse with individual PDX. The number of mice used in the E7820 treatment arms (100 mg/kg or 200 mg/kg) were three. The number of mice used in the arm without

E7820 treatment were three or four. **(b)** The relative body weight of mice during E7820 treatment. The relative tumor volume was calculated and compared to the volume of tumors on day 0.

Supplemental Fig. 1 (continued)

b

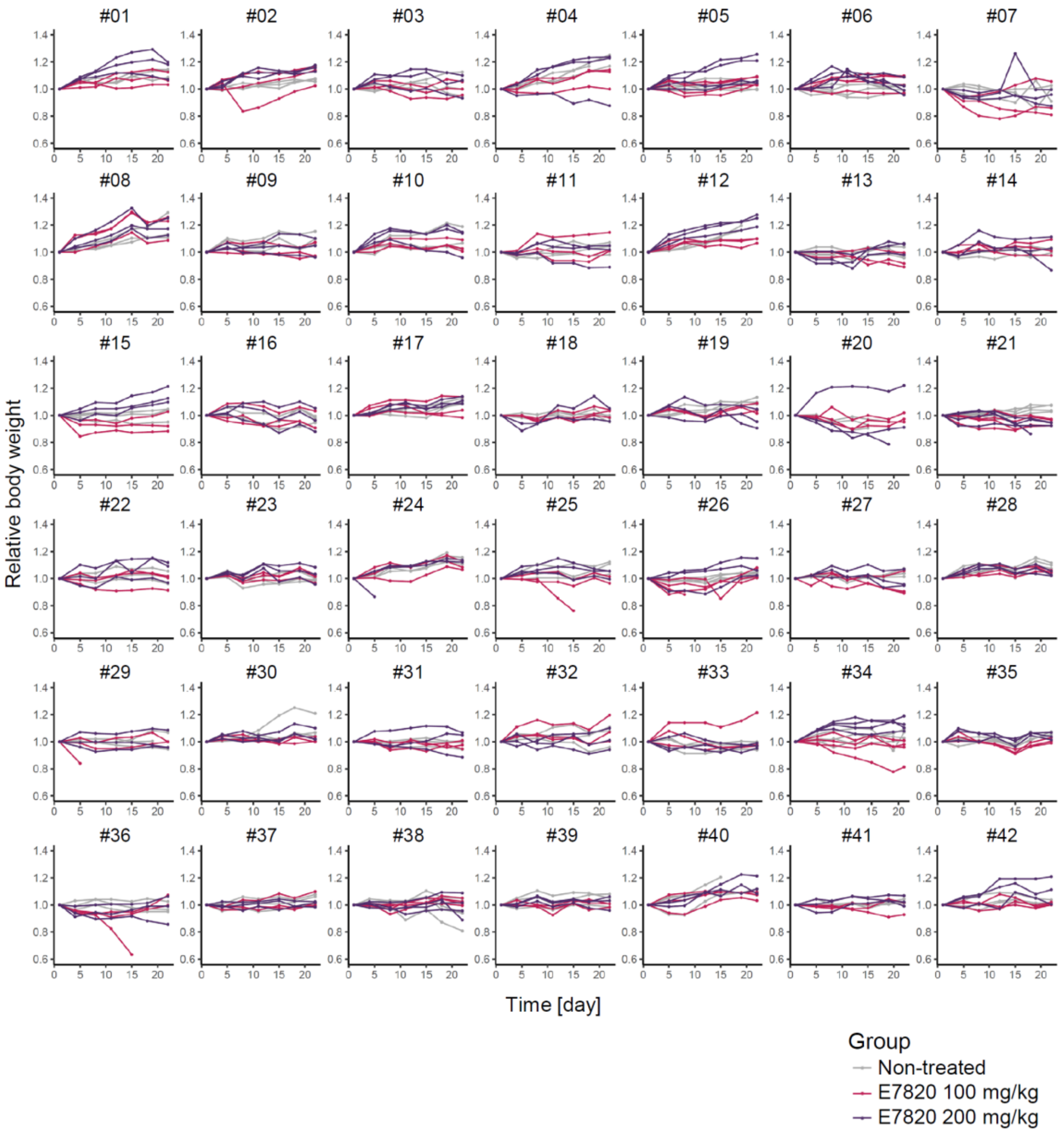

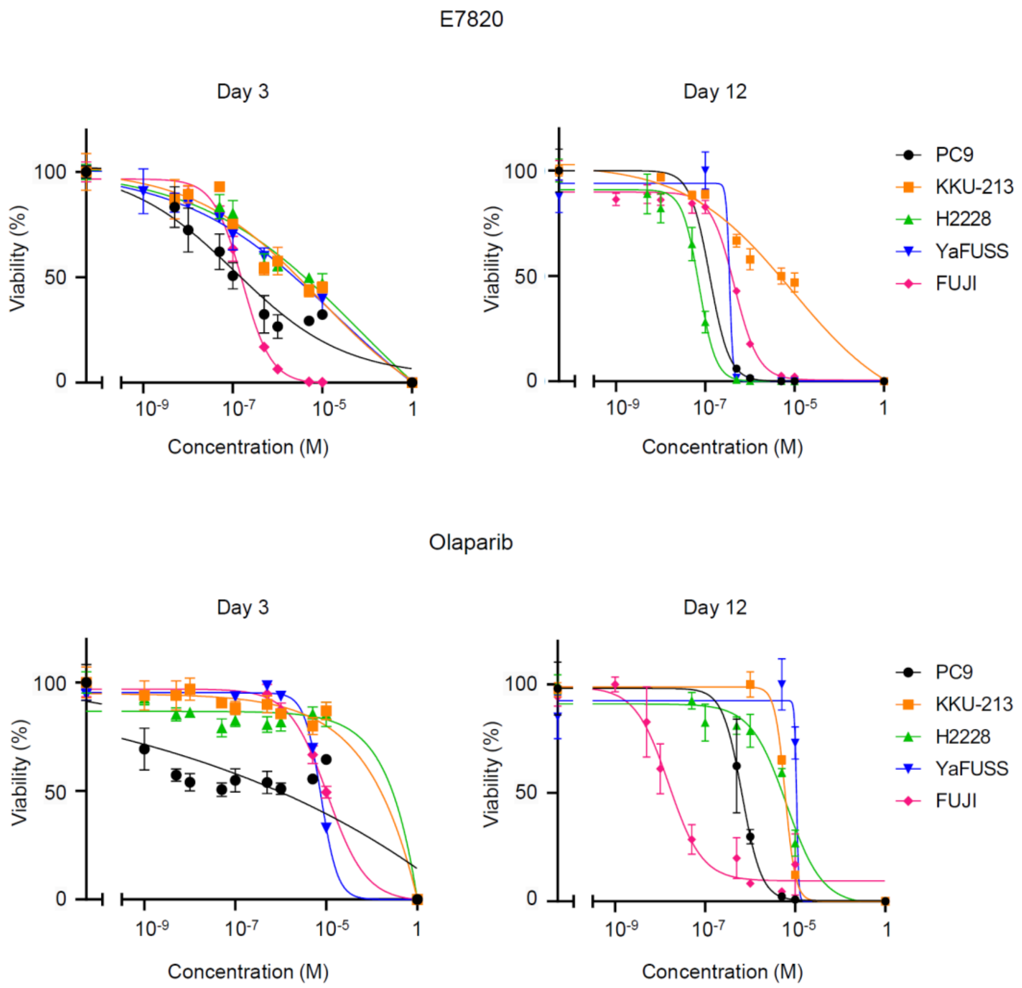

Supplemental Fig. 2. Drug efficacy evaluated in short-term or a long-term treatments of cancer cell lines.

The sensitivity of PC9, KKU-213, H2228, YaFUSS, and FUJI cells to E7820 and olaparib was investigated. Sensitivity was assessed on day 3 and 12 after the drug treatment.

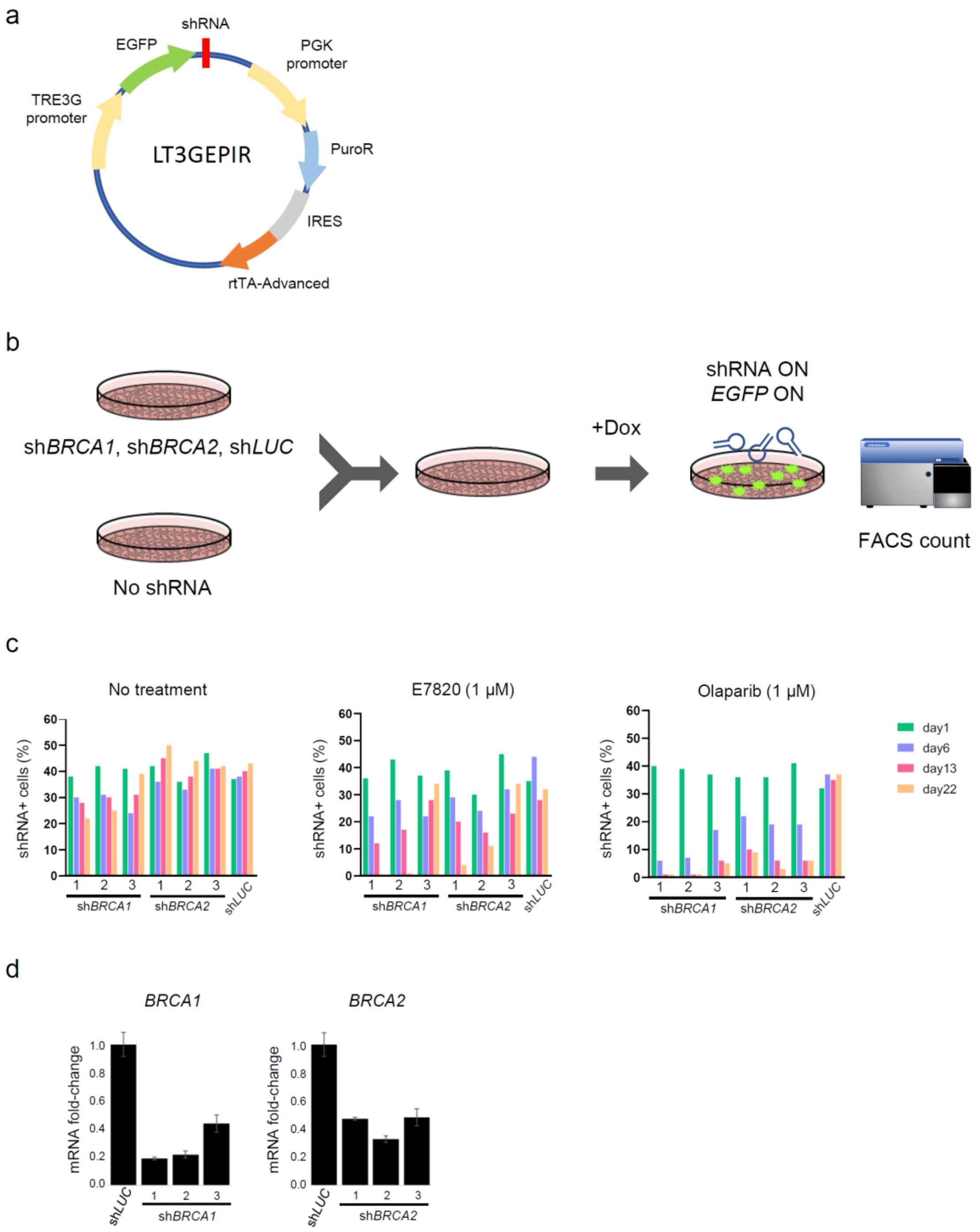

Supplemental Fig. 3. Knockdown of *BRCA1* and *BRCA2* sensitizes cells to E7820. **(a)** The map of shRNA plasmid (LT3GEPIR) for knockdown experiment. **(b)** KKU-213, an HRR-proficient cholangiocarcinoma cell line, was investigated if the knockdown of

BRCA1 or *BRCA2* could sensitize the cells to E7820. Three different shRNAs were tagged with EGFP and generated into a doxycycline-inducible expression vector to knock down individual genes. The ratio of EGFP-expressing cells with *BRCA1* or *BRCA2* shRNA was monitored temporally by FACS. **(c)** The ratio of EGFP-expressing cells gradually decreased after the cells were treated with E7820 or olaparib. As a control, shRNA for Renilla Luciferase (*LUC*) was used. **(d)** The mRNA expression of *BRCA1* and *BRCA2* was evaluated by real-time PCR. shRNA targeting *BRCA1*/*BRCA2* decreased the mRNA expression of the corresponding genes by approximately 60–80% compared to the control shRNA.

Gene	DLD1-P	DLD1-KO	PC9	FUJI	H2228	KMLS1
MRE11	0.8	0.7	0.9	0.6	0.7	1.2
RAD50	1	0.9	0.8	0.8	0.8	0.9
NBN	1.1	0.9	1	1.3	0.8	1.1
ATM	1.1	1	1.2	0.7	0.8	1.4
BRCA1	0.9	0.9	0.8	0.6	0.6	1.3
BARD1	0.6	1.2	1.9	0.6	0.6	0.7
RPA1	0.9	0.8	1	0.9	1.2	1
BRCA2	1.2	1.2	1	1.1	0.9	1.7
PALB2	0.9	0.9	1	1.1	0.8	0.9
BRIP1	0.9	0.8	1	0.9	0.8	1.2
ATR	0.6	0.6	0.6	0.7	0.5	1.2
RAD51C	1.2	1.1	0.8	1	0.9	1.2
XRCC5	1	1	1	1.1	1.1	1
XRCC6	1	1	1.1	1.2	1.1	1
PRKDC	0.9	0.8	0.7	0.7	0.8	1
DCLRE1C	1	1.2	0.8	0.8	0.6	0.9
TP53BP1	1	0.9	0.7	0.9	0.8	1
RNF168	1.2	1.1	1.2	1.3	0.9	1.1
RIF1	0.7	0.9	0.9	0.8	0.7	1.1
SHLD1	1.7	1	0.9	0.4	0.8	0.4
SHLD2	0.8	0.8	0.7	0.7	0.5	1
SHLD3	0.8	0.8	1.3	1.4	0.7	0.6
MAD2L2	1	1	0.9	0.9	1	0.9
XRCC4	1.2	1	1	1.2	1	1
NHEJ1	1.6	1.4	1.4	1.3	0.8	1.1
LIG4	1	1.2	1.3	1.3	0.9	1

Gene	DLD1-P	DLD1-KO	PC9	FUJI	H2228	KMLS1
FANCA	0.8	1	0.9	0.5	0.6	0.9
FANCB	1.1	1.3	1.1	0.8	0.9	1.2
FANCC	1.5	1.3	1.3	1.3	1.2	1.1
FANCD2	0.6	0.6	0.9	0.6	0.5	0.7
FANCE	0.8	0.8	0.9	0.8	0.5	0.9
FANCF	1	1	0.9	1.2	0.9	1.2
FANCG	0.8	0.8	0.8	0.4	0.7	1
FANCI	0.6	0.7	0.6	0.4	0.5	0.8
FANCL	0.7	0.8	0.8	0.6	0.5	1
FANCM	1.1	1.3	1	1.7	1.4	1
UBE2T	1	0.9	0.8	1.1	1.2	1.1
CENPS	0.6	0.8	0.7	0.3	0.5	1
CENPX	1.1	1	0.9	0.6	0.9	0.9
FAAP20	1	1	0.9	0.7	0.8	1
FAAP24	1.2	1	0.9	1	1.1	1.2
FAAP100	1	0.9	0.6	0.5	0.7	1.2
UHRF1	0.8	0.8	0.6	0.7	1	1.1
SLX1A	0.5	0.6	0.4	0.6	0.5	0.9
SLX4	0.8	1	0.9	1.1	1	1
FAN1	0.8	0.8	0.8	0.9	0.8	1.1
XPC	0.7	0.8	0.9	0.4	0.6	1.2
CETN2	1.1	1	1.2	1.1	1.2	1
RAD23B	1.1	1	0.9	1.2	0.9	1
UVSSA	0.8	0.8	1	0.6	0.8	1.3
DDB1	1	0.9	0.8	0.7	1	1
DDB2	0.6	0.5	0.7	1.2	0.6	0.8
ERCC1	0.8	1	0.8	0.7	0.9	1.1
ERCC2	0.8	0.8	0.9	0.6	0.8	1.2
ERCC3	0.9	0.9	1.1	1.1	1.2	1.2
ERCC4	1.2	1	0.9	1.1	1.1	0.9
ERCC5	0.8	0.8	0.5	0.6	0.7	1
ERCC6	1	0.9	0.9	1.2	0.6	1
ERCC8	1	1	0.7	0.9	0.9	0.9
GTF2H1	0.8	0.8	0.7	0.4	0.4	1
GTF2H2	1.2	1	1.6	1.4	0.9	1.1
GTF2H3	1	1.2	1	0.9	1.1	0.9
GTF2H4	0.9	0.8	0.8	0.7	0.9	1.1
GTF2H5	1.1	1.2	1.1	1.2	1.5	0.9
XPA	1.3	1.5	1	0.9	0.9	1.1

Gene	DLD1-P	DLD1-KO	PC9	FUJI	H2228	KMLS1
APEX1	1	1	1	0.9	1.1	0.9
PARP1	1	1	0.9	1.1	1	0.9
PARP2	1.4	1.2	1.3	1.3	1.1	1.1
PARP3	0.9	1.3	0.8	1	0.8	1
PARP6	0.9	0.9	1.2	1	0.9	1.2
PARP9	1	1.3	1	0.5	1	1.1
PARP14	1	1.1	0.8	0.7	0.8	0.9
XRCC1	0.7	0.7	0.6	0.7	0.7	1
BLM	1	1.2	1.3	1.1	1	1.2
CCNH	0.4	0.4	0.4	0.5	0.3	0.7
CDK7	1.2	1.1	1.3	1.4	0.9	1
DNA2	1	0.8	1	0.6	0.7	0.6
EXO1	1	1	1.1	0.5	0.7	0.7
LIG1	1	0.9	0.8	0.6	0.8	0.9
LIG3	0.8	0.7	0.6	0.8	0.5	0.9
MNAT1	1	1.1	0.8	0.8	0.9	1
PCNA	0.9	0.9	0.8	0.6	0.8	0.9
POLD1	1.1	1	0.8	0.6	1	1
POLD2	1	0.9	0.9	0.6	0.8	0.9
POLD3	0.7	0.7	0.8	0.5	0.7	0.7
POLD4	1.1	1.3	0.9	0.8	1	0.8
POLE2	0.6	0.7	0.9	0.7	0.5	1
POLE3	1.1	1	1	1	1.2	1
POLK	1	1.1	0.8	1.3	0.7	1.2
REV1	1.1	0.9	1	0.9	0.9	1.3
TOP1	1	0.9	1.1	1.1	0.7	1
TOP2A	0.7	0.7	0.5	0.3	0.3	0.9
TOP2B	0.8	0.8	0.7	0.7	0.6	0.8

HR
NHEJ
FANC
NER
BER
Common

Supplemental Fig. 4. The changes in gene expression induced by E7820 treatment. Fold changes are shown in a heatmap comparing the expression levels in cell lines after E7820 treatment (1 µM for 48 h) to basal expression. These genes are involved in homologous recombination repair (HRR), non-homologous end joining (NHEJ), Fanconi anemia complementation group (FANC) pathway, nucleotide excision repair

(NER), base excision repair (BER), and other pathways related to DNA damage repair (Common).

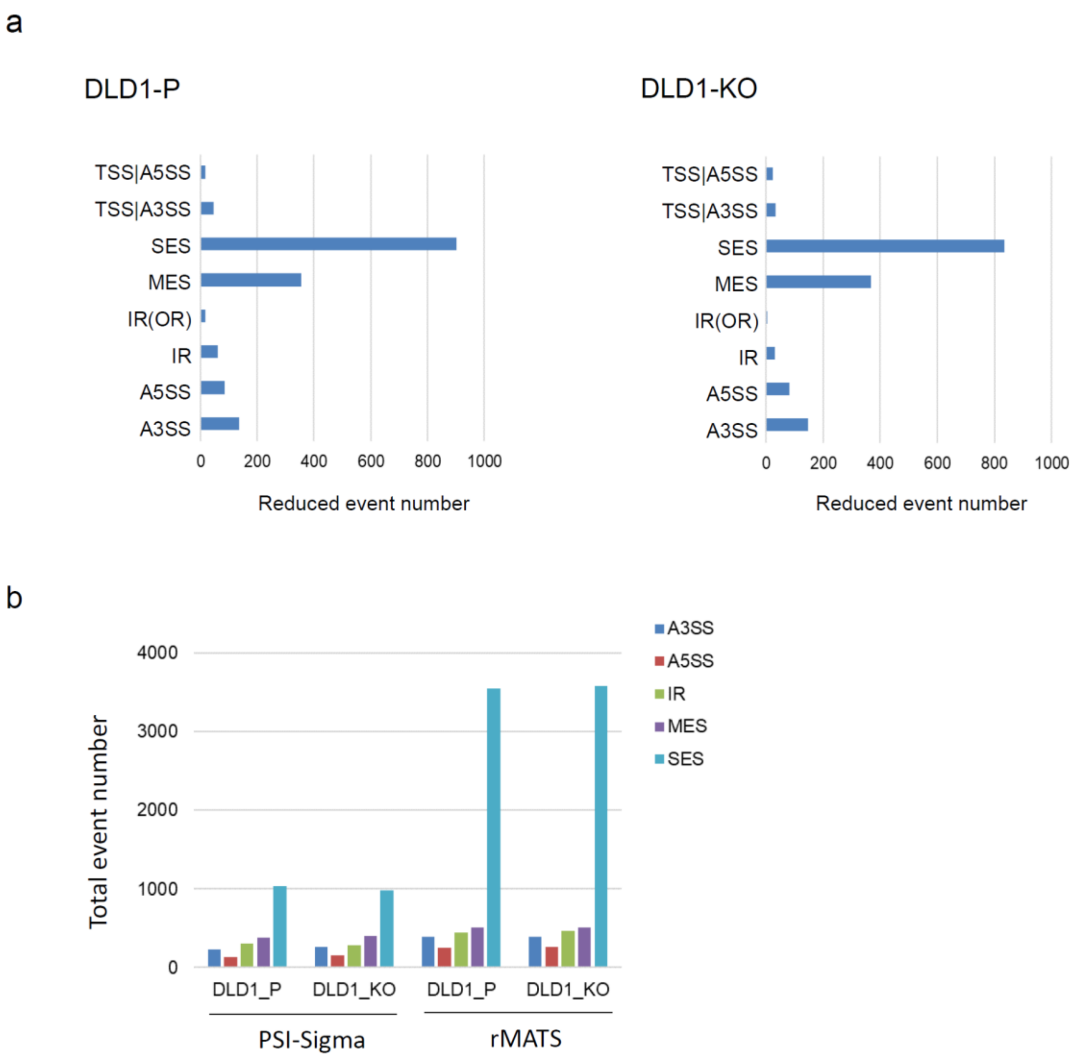

Supplemental Fig. 5. Alternative splicing events changed by E7820 treatment. **(a)** The number of splicing anomalies reduced by E7820 treatment are indicated. Similar alternative splicing events were induced by E7820 in both DLD1-P and DLD1-KO cells. Mis-splicing event codes are as follows: TSS, transcription start site; A5SS, alternative 5’ splice site; A3SS, alternative 3’ splice site; SES, single-exon skipping; MES, multiple-exon skipping; IR, intron retention; OR, overlapping region. **(b)** The

comparison of PSI-sigma and rMATS. The total (induced and reduced) alternative splicing event were evaluated by PSI-sigma and rMATS.

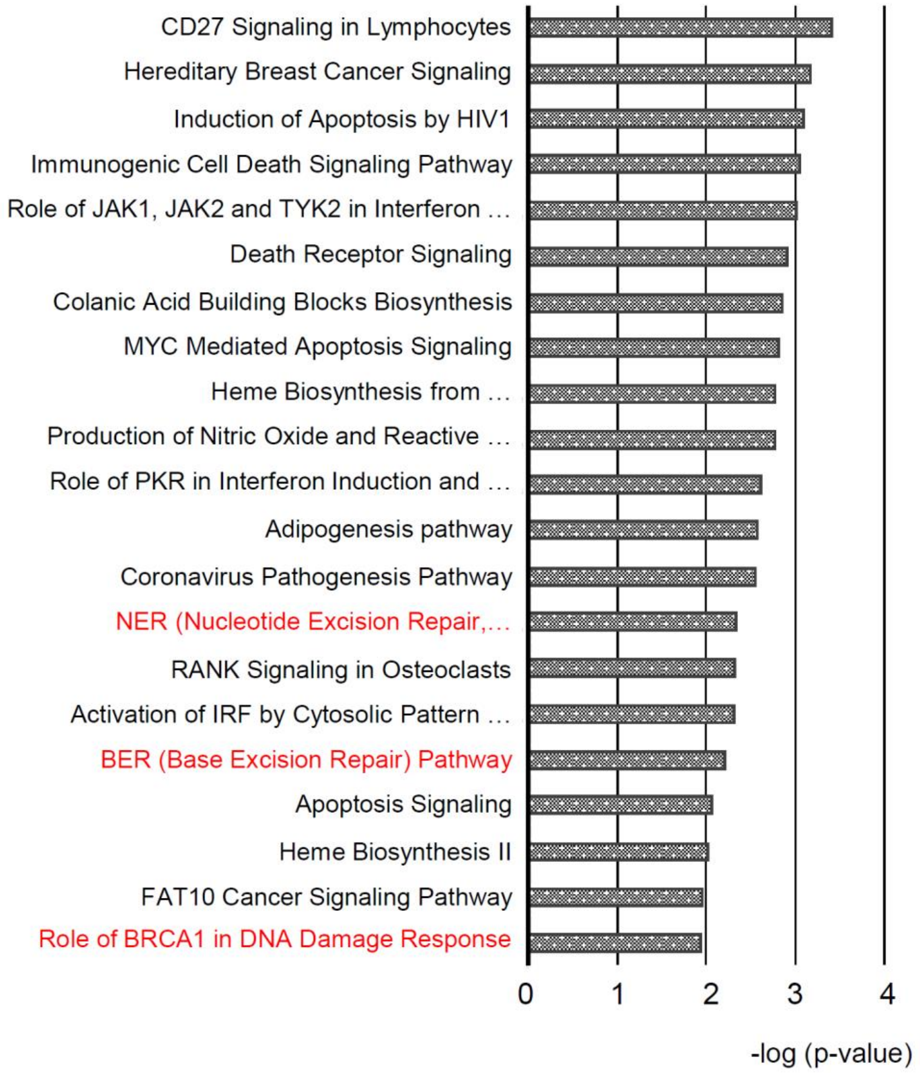

Supplemental Fig. 6. Pathway analysis of genes induced intron retention by E7820. Intron retention induced by E7820 in more than two cell lines was observed in 412 genes. These genes were subjected to IPA, revealing that the shared pathways among them included “Hereditary Breast Cancer Signalling” and “Role of BRCA1 in DNA Damage Response”.

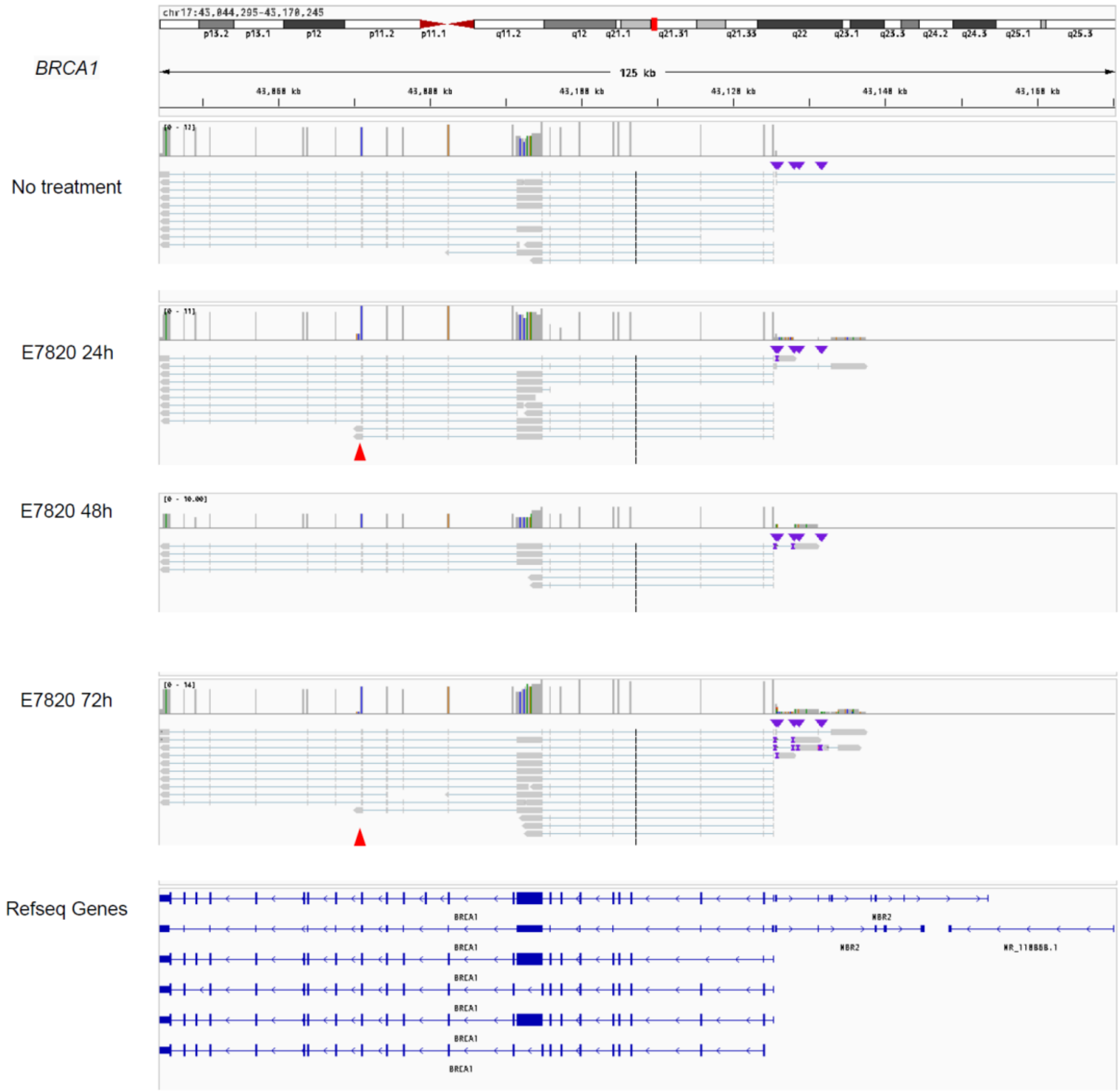

Supplemental Fig. 7. The novel intron retentions in genes related to DNA damage repair.

To confirm the characteristics of the transcripts in E7820-treated cells, full-length cDNAs were selectively prepared from the cells and sequenced using a Sequel II long-read sequencer (Pacific Biosystems). The long-read sequences were used to identify isoforms using the bulk Iso-seq and pigeon workflows. The isoforms were visualized

using Integrative Genomics Viewer (IGV). Novel intron retentions in *BRCA1*, *FANCA*, *FANCD2*, *ERCC5*, *FAN1*, *FANCG*, *FANCI*, *XPC* induced by E7820 treatment are indicated with arrowheads.

Supplemental Fig. 7 (continued)

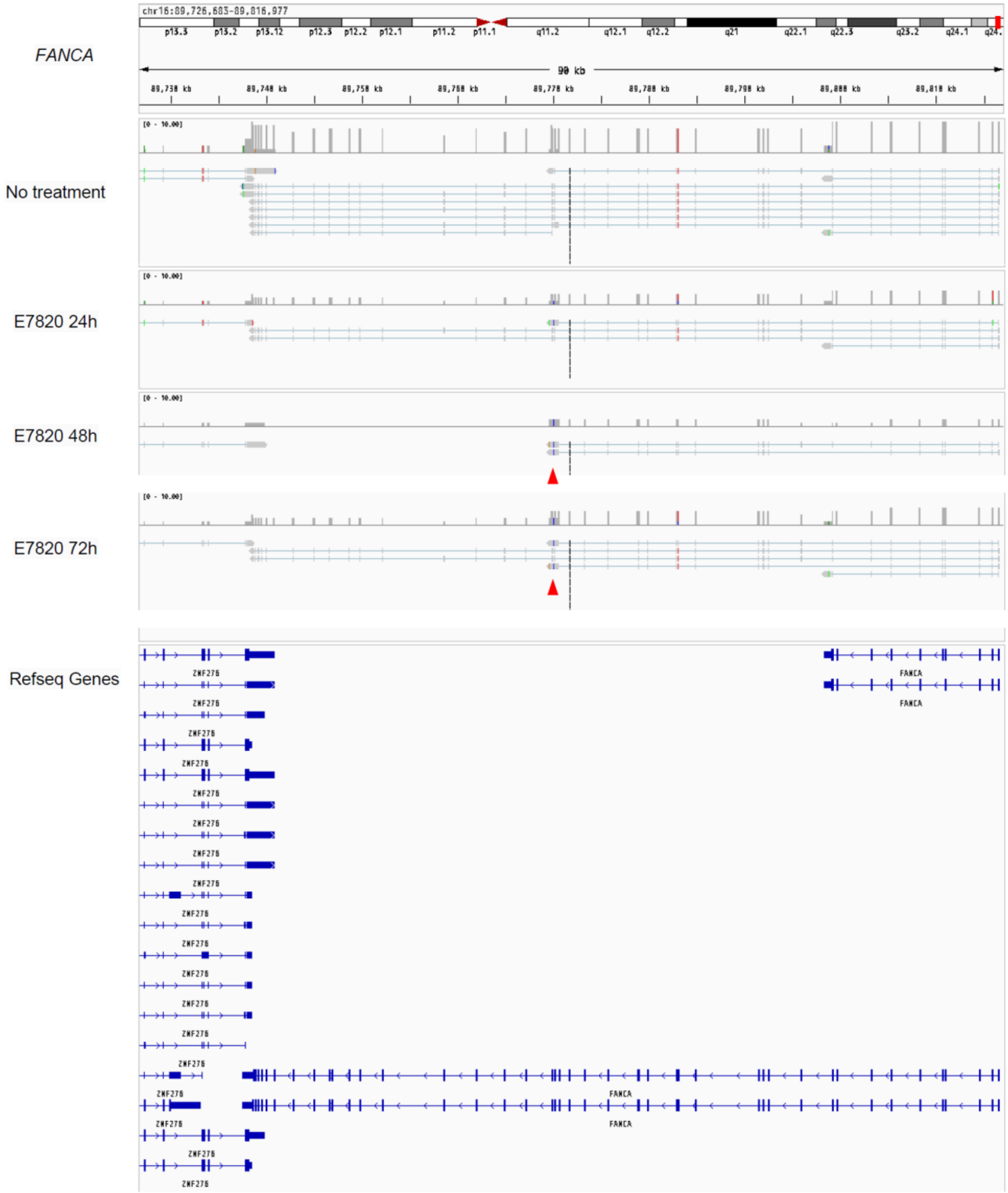

Supplemental Fig. 7 (continued)

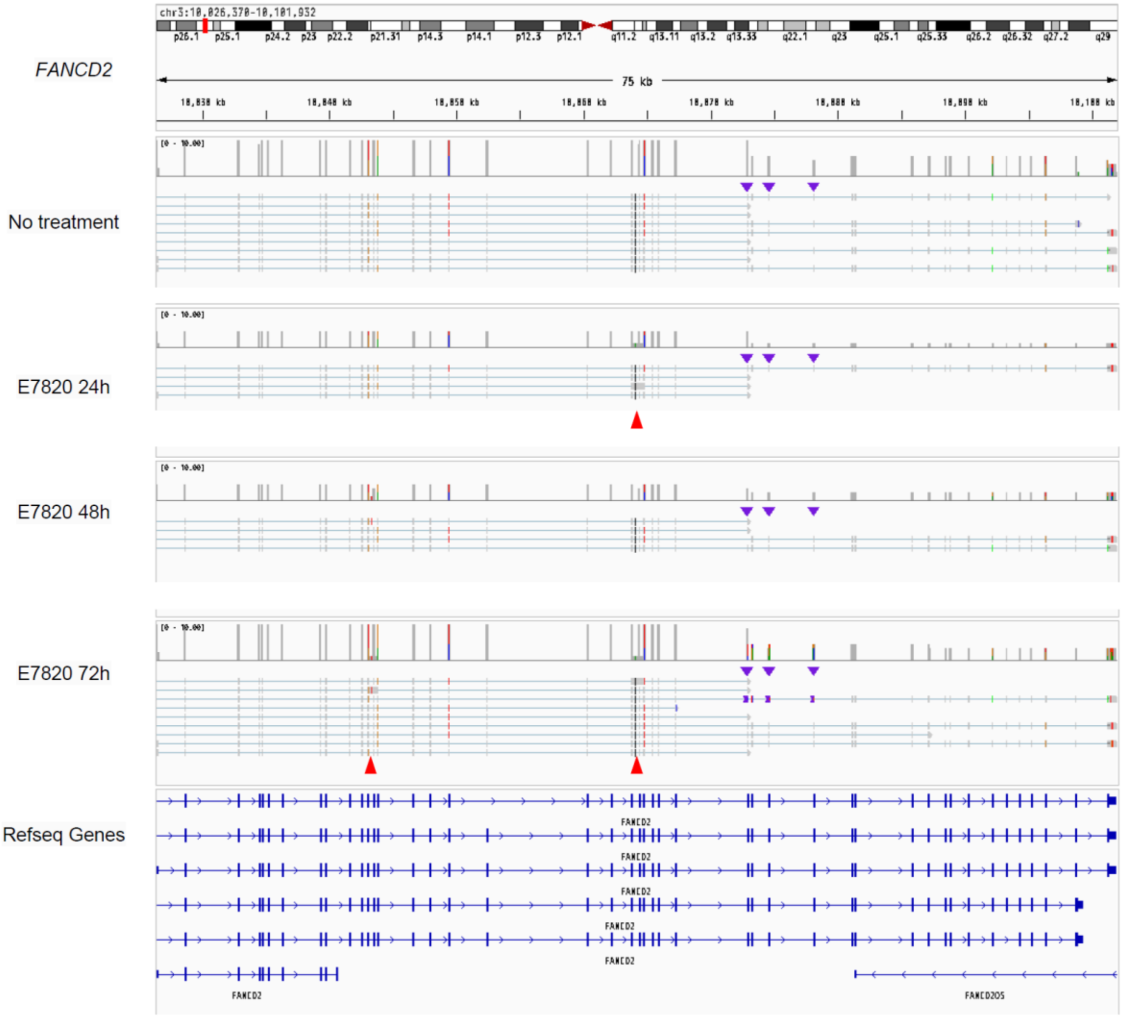

Supplemental Fig. 7 (continued)

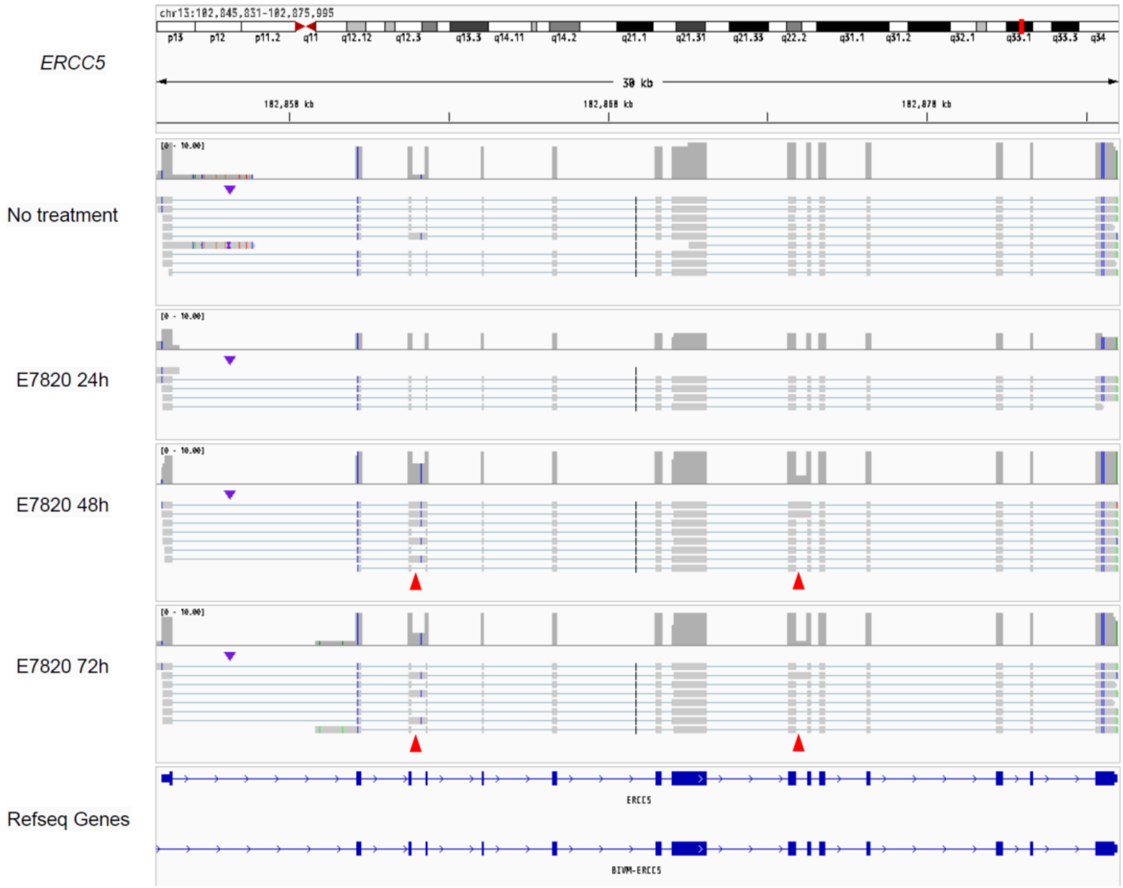

Supplemental Fig. 7 (continued)

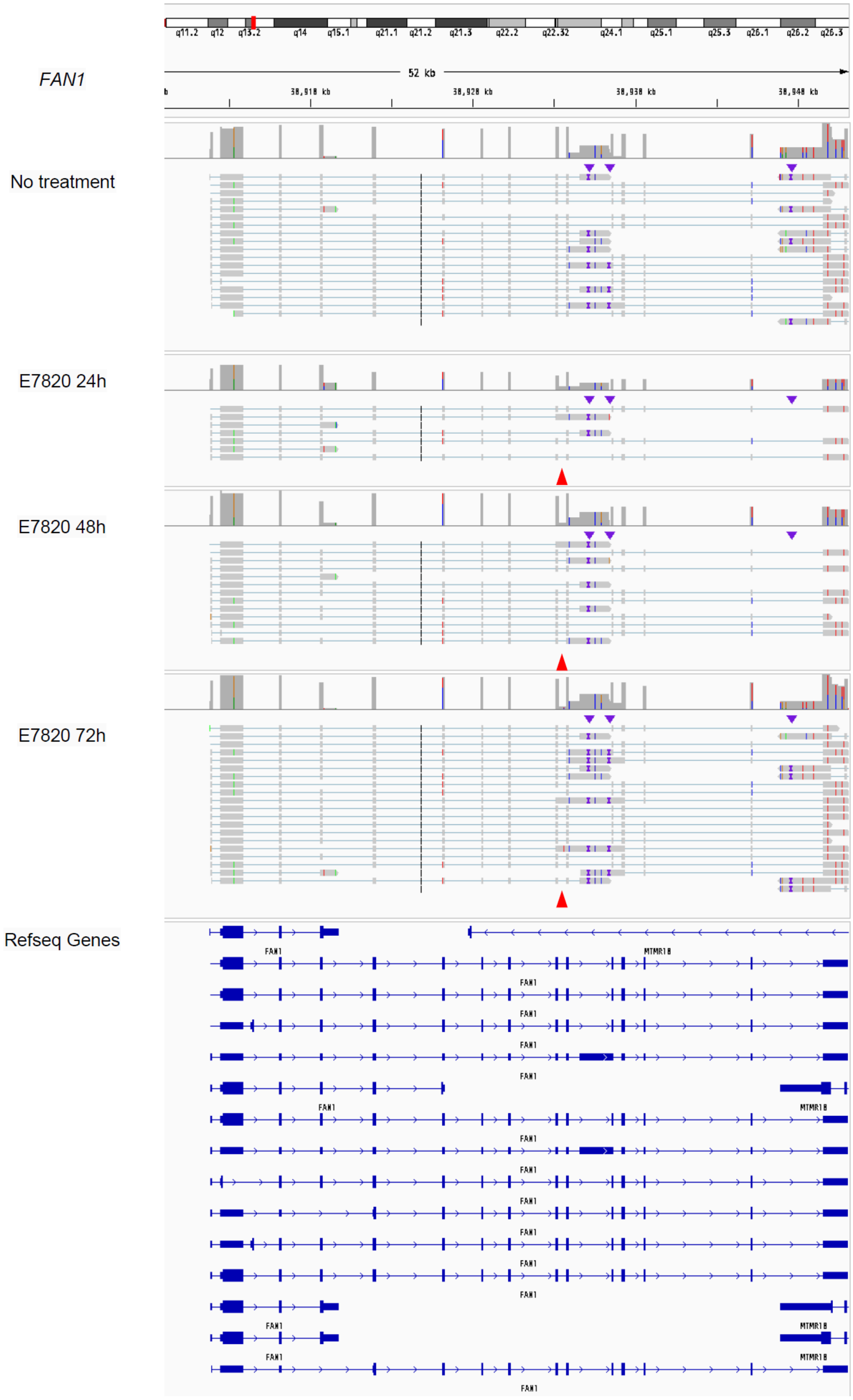

Supplemental Fig. 7 (continued)

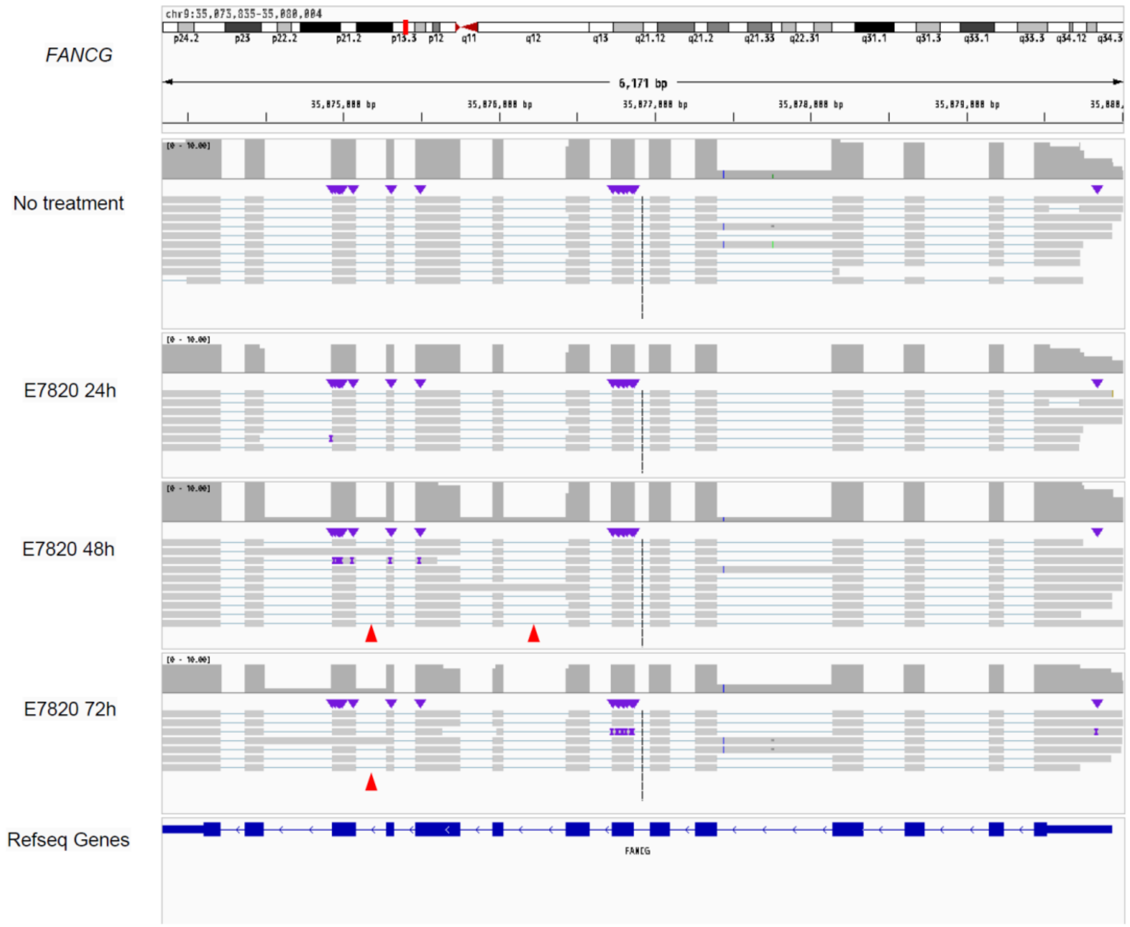

Supplemental Fig. 7 (continued)

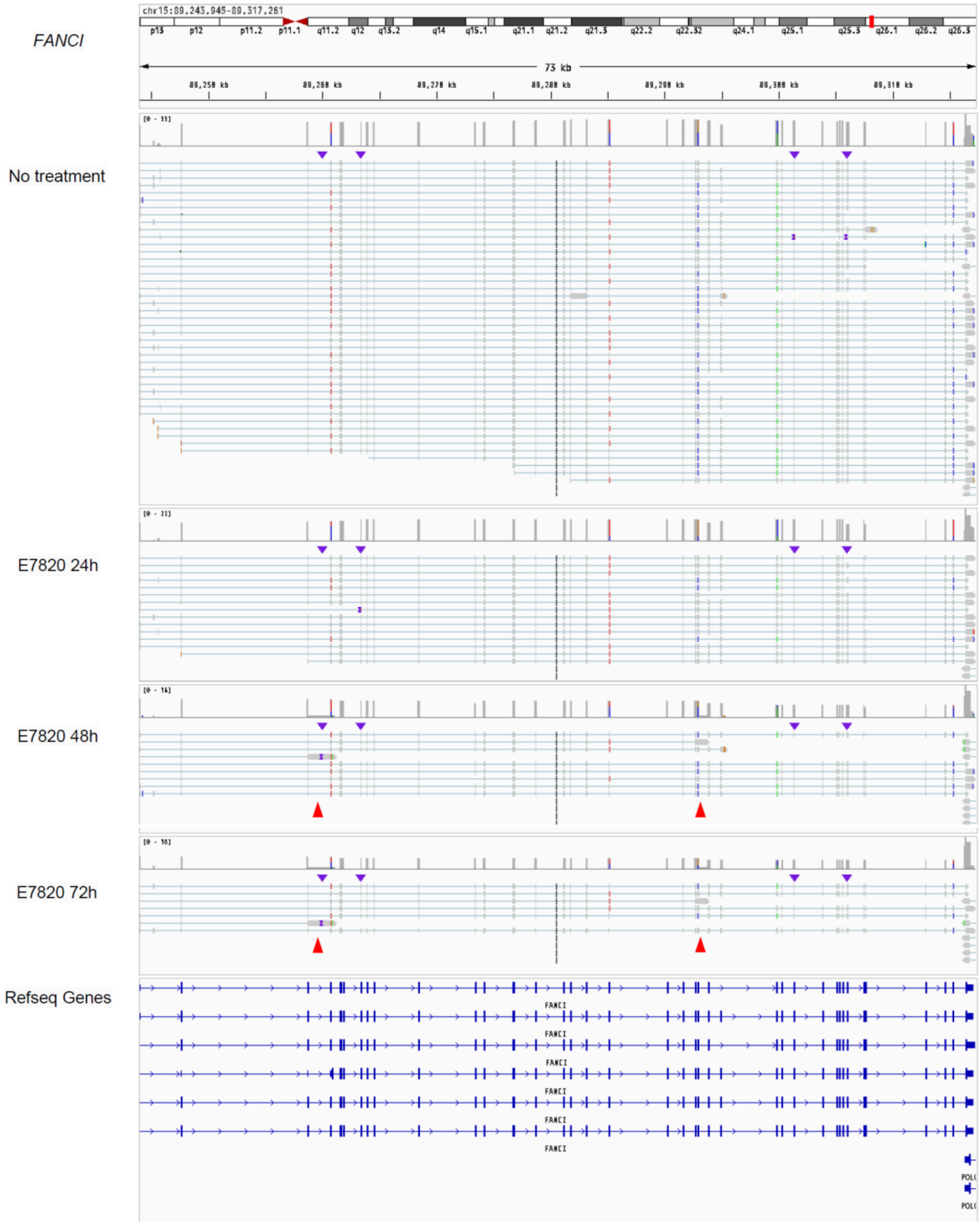

Supplemental Fig. 7 (continued)

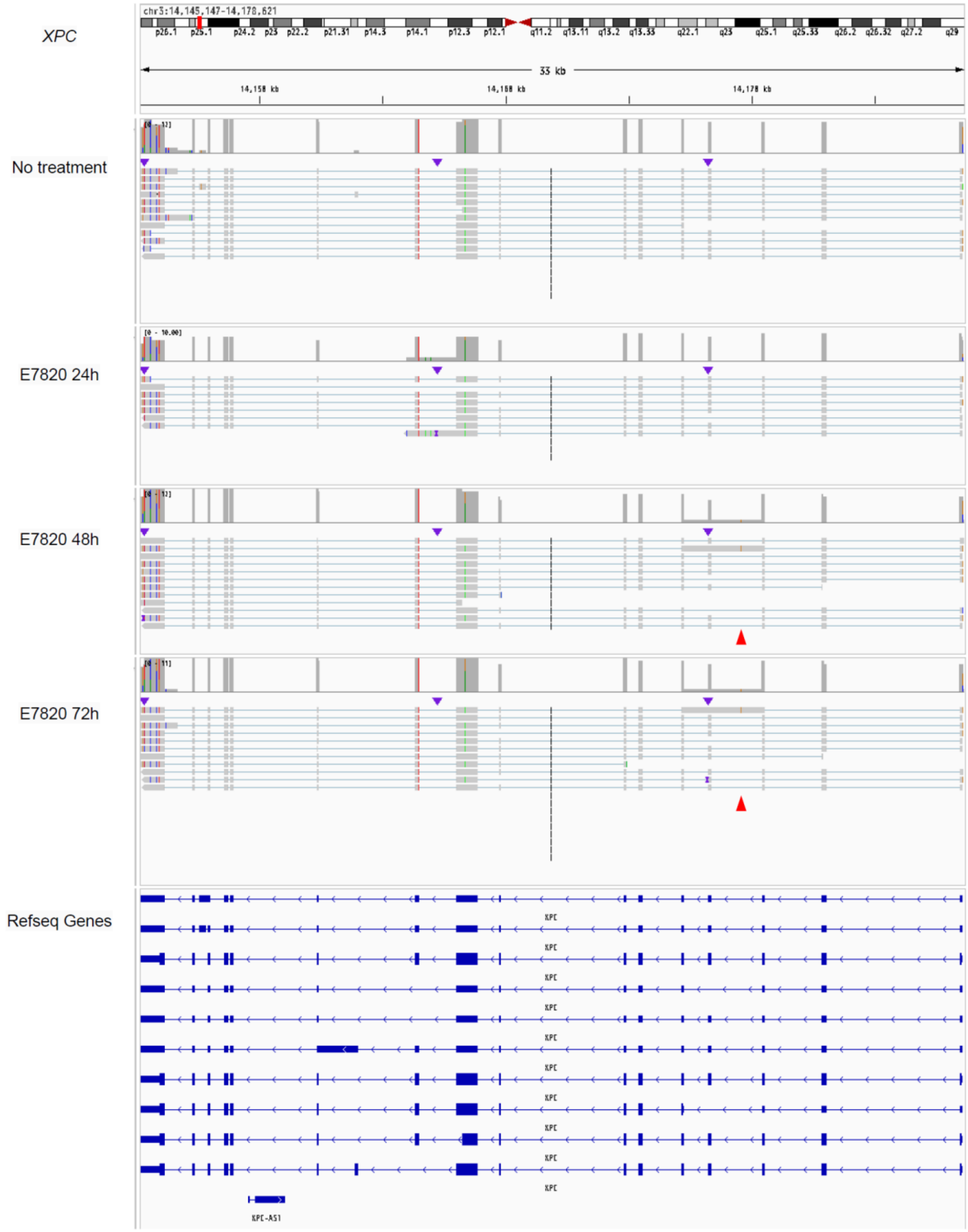

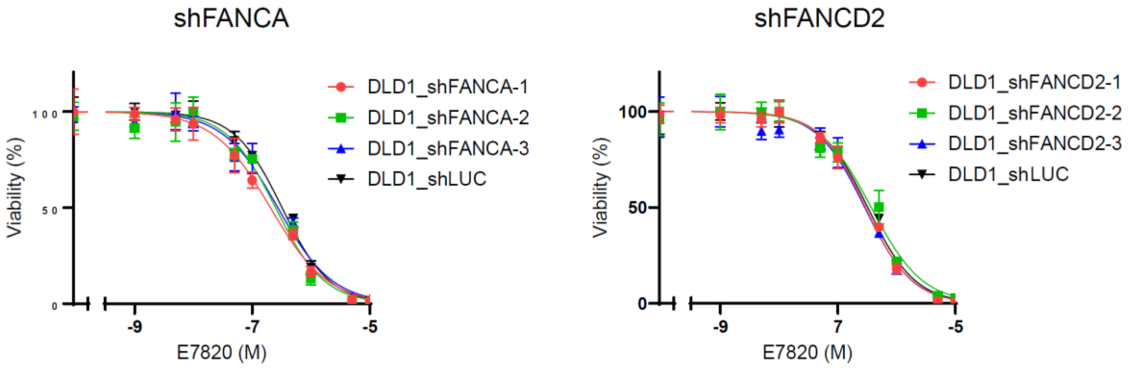

IC_{50} (E7820, μM)

	FANCA	FANCD2	LUC
shRNA #1	0.20	0.30	0.32
shRNA #2	0.25	0.37	NA
shRNA #3	0.27	0.29	NA

Supplemental Fig. 8. Knockdown of *FANCA* or *FANCD2* sensitizes cells to E7820. DLD1-P, an HRR-proficient human colon cancer cell line was investigated if the knockdown of *FANCA* or *FANCD2* could sensitize the cells to E7820. Three different shRNAs were tagged with EGFP and generated into a doxycycline-inducible expression vector to knock down individual genes. The ratio of EGFP-expressing cells with *FANCA* or *FANCD2* shRNA was monitored temporally by FACS. The ratio of EGFP-expressing cells gradually decreased after the cells were treated with E7820 or olaparib. As a control, shRNA for Renilla Luciferase (LUC) was used.

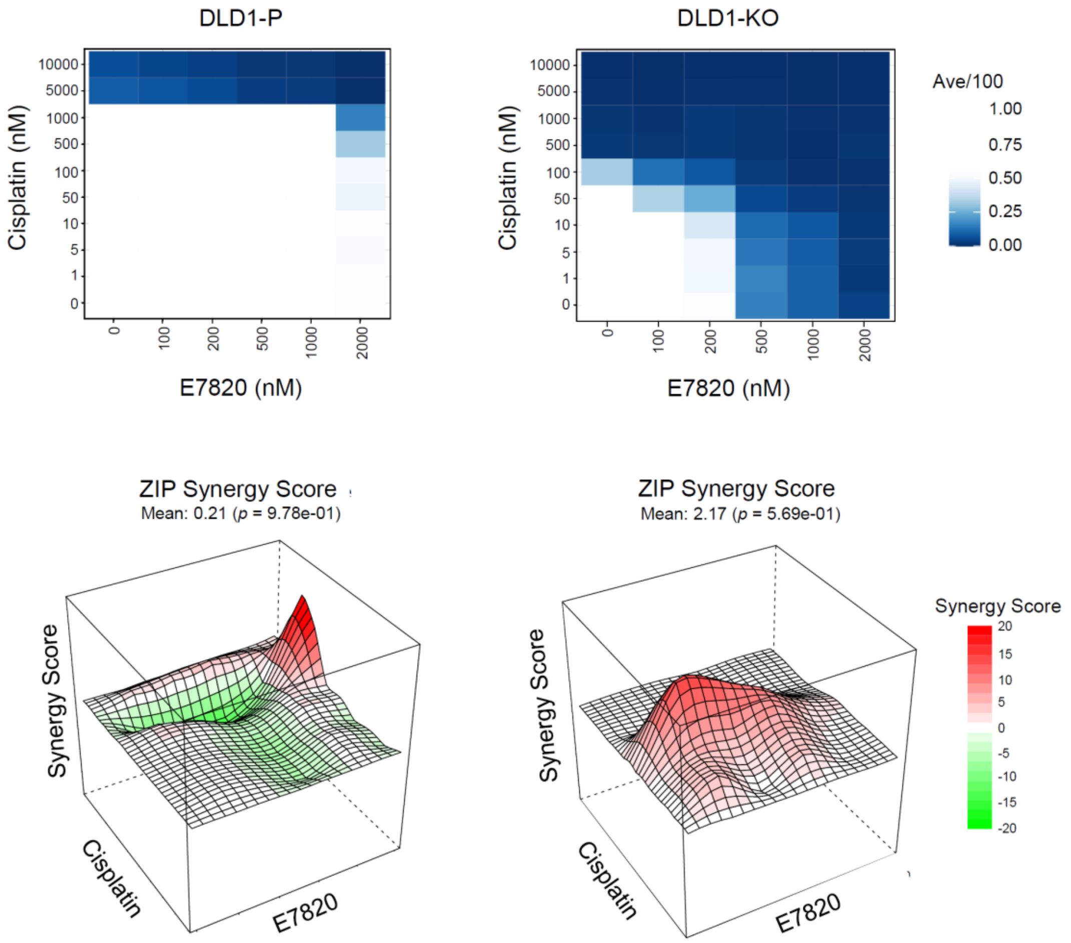

Supplemental Fig. 9. Combination therapy with E7820 and cisplatin.

DLD1-P and DLD1-KO cells were treated with a combination of E7820 and cisplatin at indicated concentrations. Cell viability was assessed using the PrestoBlue cell viability assay and is indicated with color-coded matrices. Synergistic effects are indicated in the 3D drug synergy maps. The surfaces of the maps were color-coded according to the ZIP scores of the combination treatment. A mild synergistic effect was observed around the IC_{50} in DLD1-KO cells.

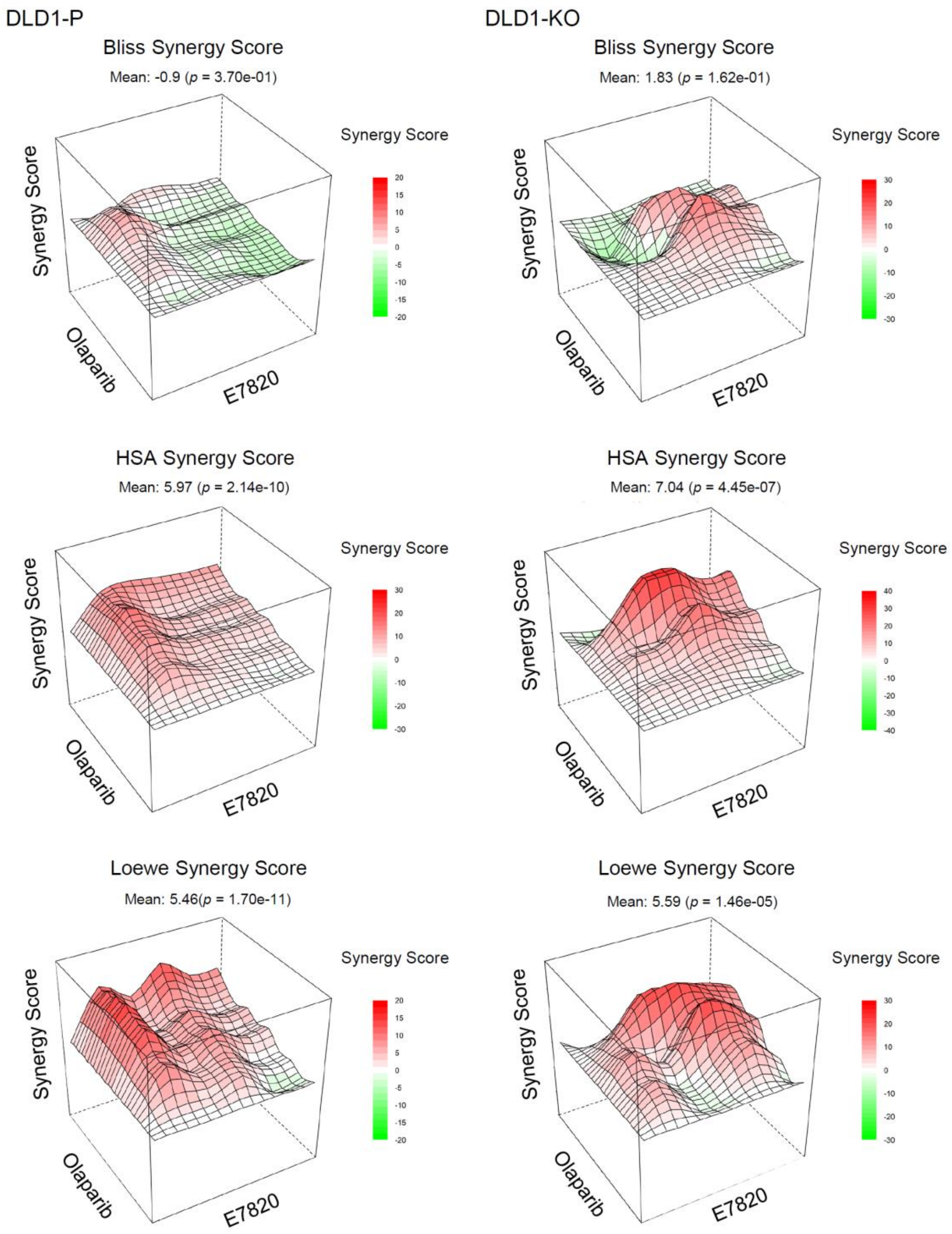

Supplemental Fig. 10. Combination therapy with E7820 and olaparib.

DLD1-P and DLD1-KO cells were treated with a combination of E7820 and olaparib at indicated concentrations. Cell viability was assessed using the PrestoBlue cell viability

assay. Synergistic effects are indicated in the 3D drug synergy maps. The surfaces of the maps were color-coded according to the synergy scores (Bliss, HSA and Loewe) of the combination treatment. A mild synergistic effect was observed around the IC_{50} in DLD1-KO cells.

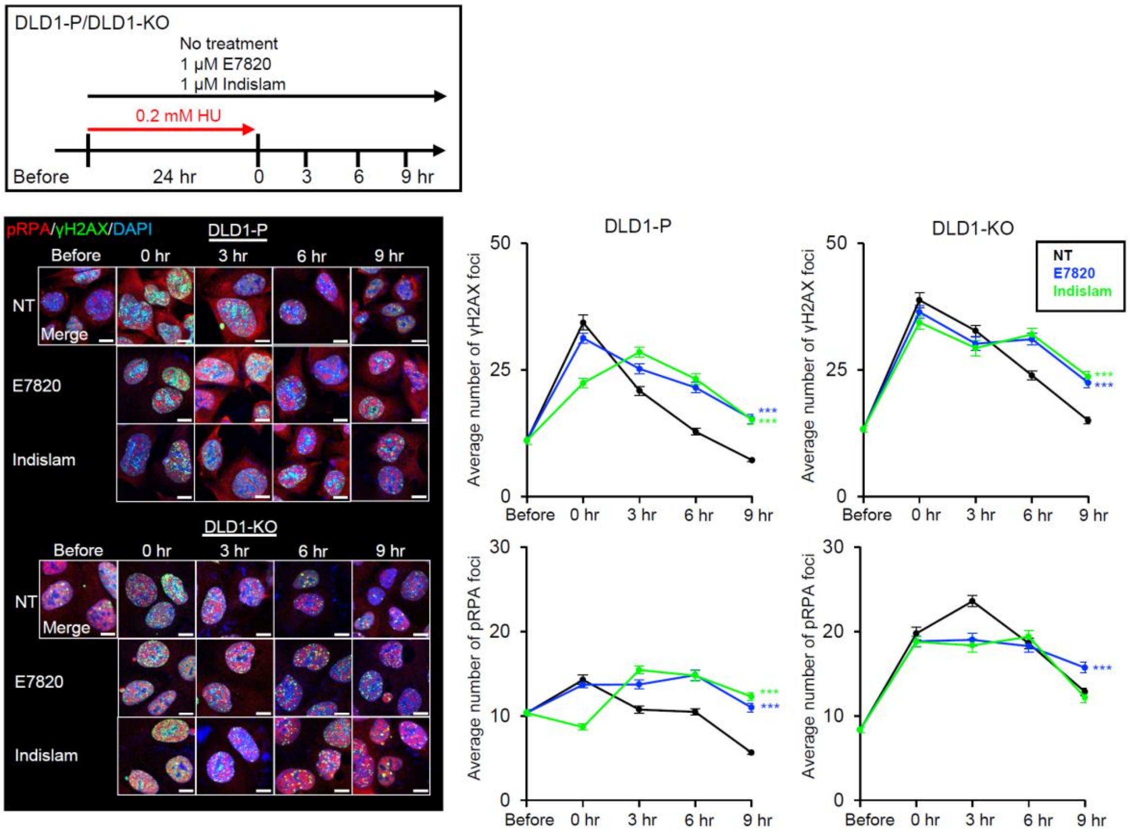

Supplemental Fig. 11. The attenuation of repair competence induced by E7820.

DLD1 cells (P or KO) were treated with E7820 (1 µM) or indisulam (1 µM) during and after hydroxyurea (0.2 mM) treatment, and γH2AX and pRPA foci were analyzed. Representative images are shown here. Scale bars in the images, 10 µm. Dots and error bars in the graph show means ± standard errors. Two-tailed Welch's t-test was used for statistical analysis. NT, no treatment.

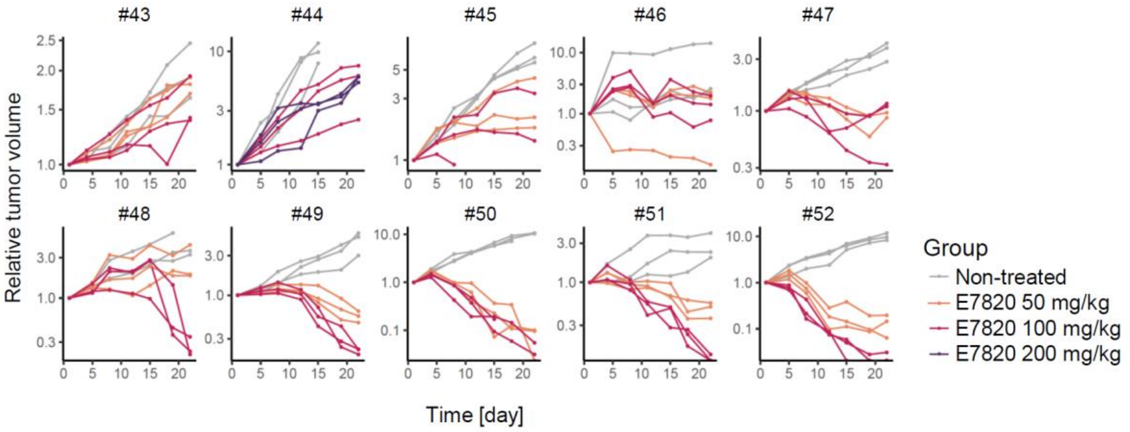

Supplemental Fig. 12. The evaluation of E7820 efficacy in PDX models involving mutations in HRR genes.

The growth curves of the E7820-treated PDXs are shown in **Fig. 4d**. Each line indicates one tumor. The relative tumor volume was calculated and compared to the volume of tumors on day 0.

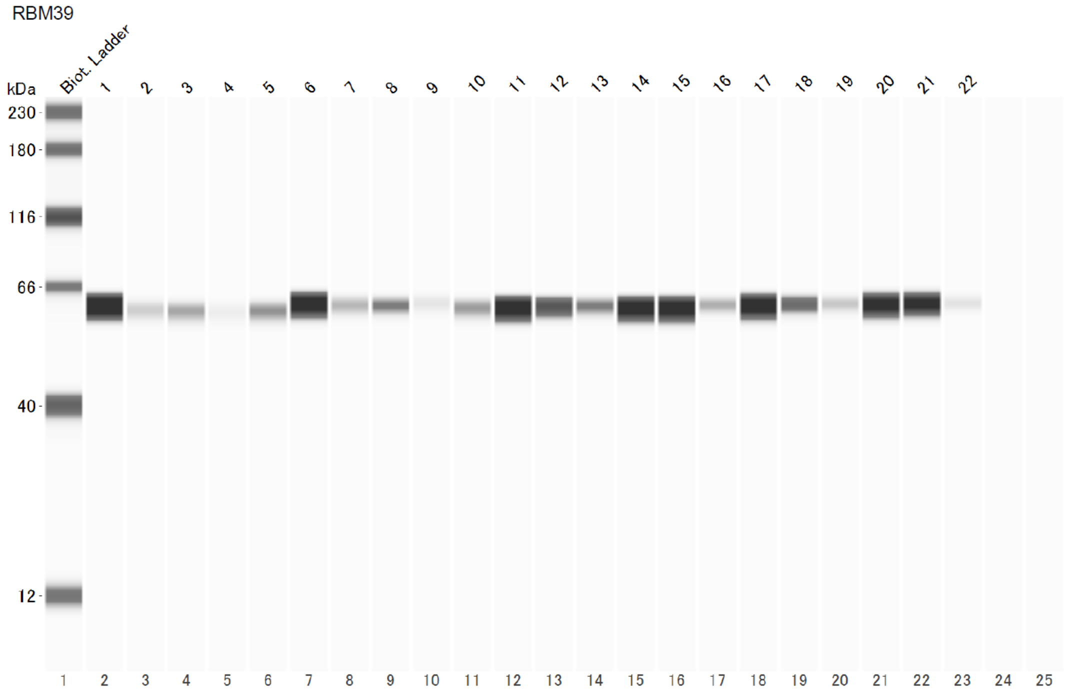

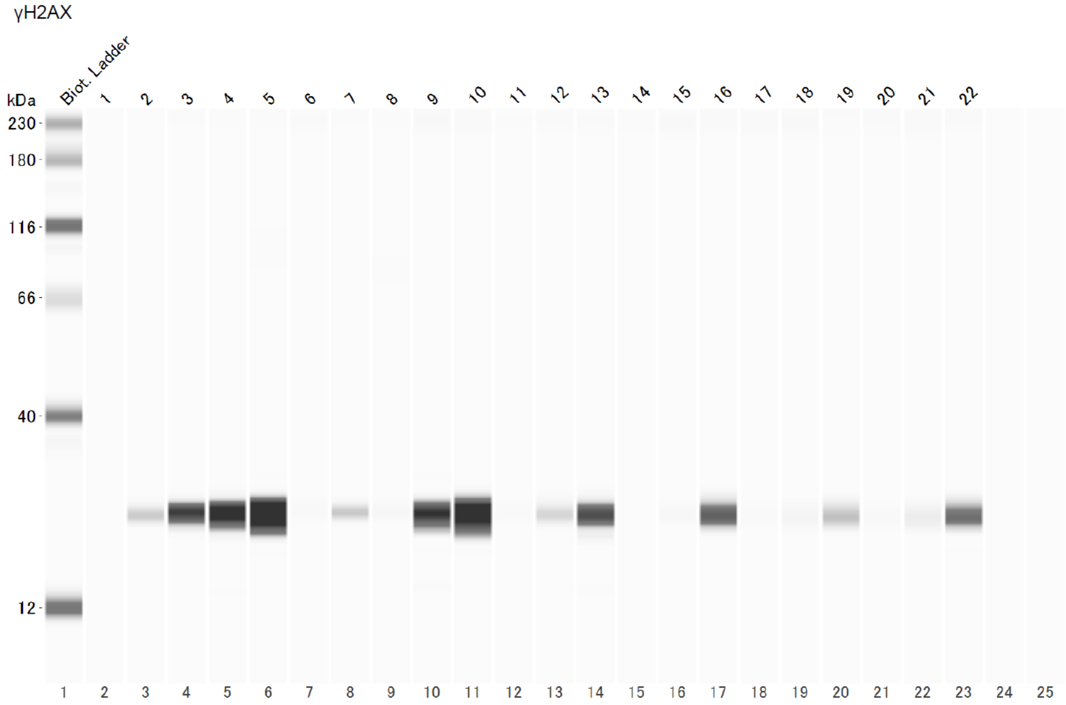

Supplemental Fig. 13. Uncropped images of western blotting

The uncropped images of western blot of **Fig. 2a, 2b, 3g and 4c**.

Supplemental Fig. 13 (continued)

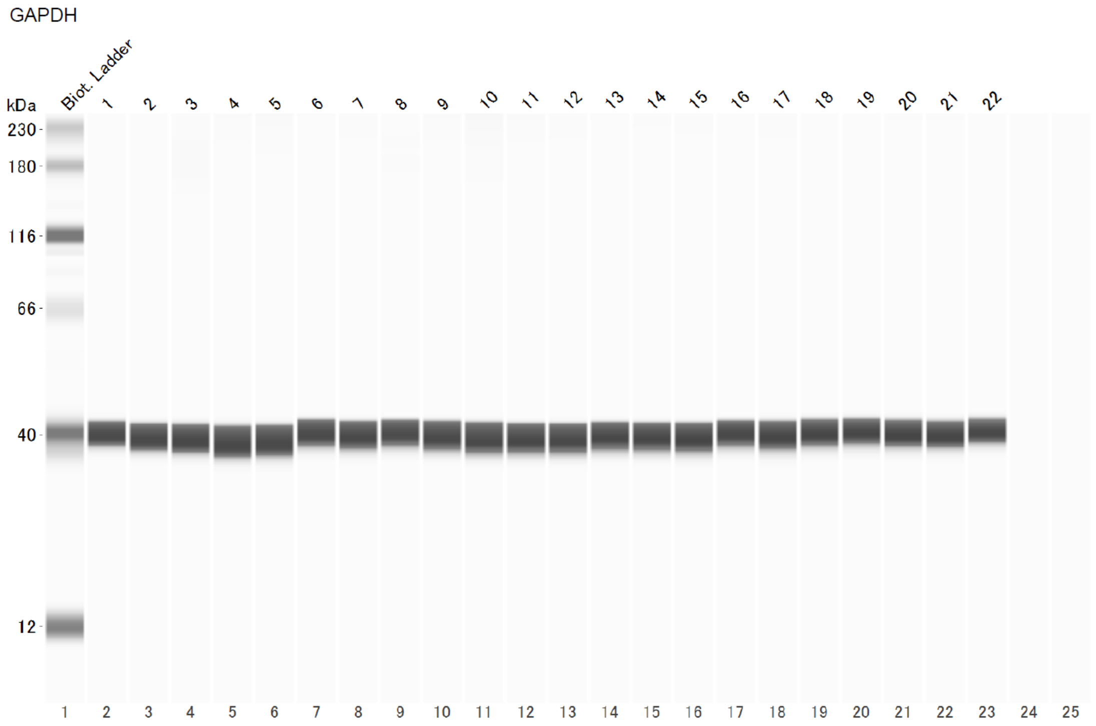

Lane	Cell	Time point	Drug
1	DLD1-KO	0 hrs	No treatment
2	DLD1-KO	24hrs	E7820 1μM
3	DLD1-KO	48hrs	E7820 1μM
4	DLD1-KO	72hrs	E7820 1μM
5	DLD1-KO	120hrs	E7820 1μM
6	DLD1-P	0 hrs	No treatment
7	DLD1-P	24hrs	E7820 1μM
8	DLD1-P	48hrs	E7820 1μM
9	DLD1-P	72hrs	E7820 1μM
10	DLD1-P	120hrs	E7820 1μM
11	DLD1-P	0 hrs	No treatment
12	DLD1-KO	24hrs	E7820 0.1μM
13	DLD1-KO	48hrs	E7820 1μM
14	DLD1-KO	72hrs	Olaparib 0.1μM
15	DLD1-KO	120hrs	Olaparib 1μM
16	DLD1-KO	72hrs	E7820 1μM + Olaparib 1μM
17	DLD1-P	72hrs	No treatment
18	DLD1-P	72hrs	E7820 0.1μM
19	DLD1-P	72hrs	E7820 1μM
20	DLD1-P	72hrs	Olaparib 0.1μM
21	DLD1-P	72hrs	Olaparib 1μM
22	DLD1-P	72hrs	E7820 1μM + Olaparib 1μM

Supplemental Fig. 13 (continued)

FANCD2

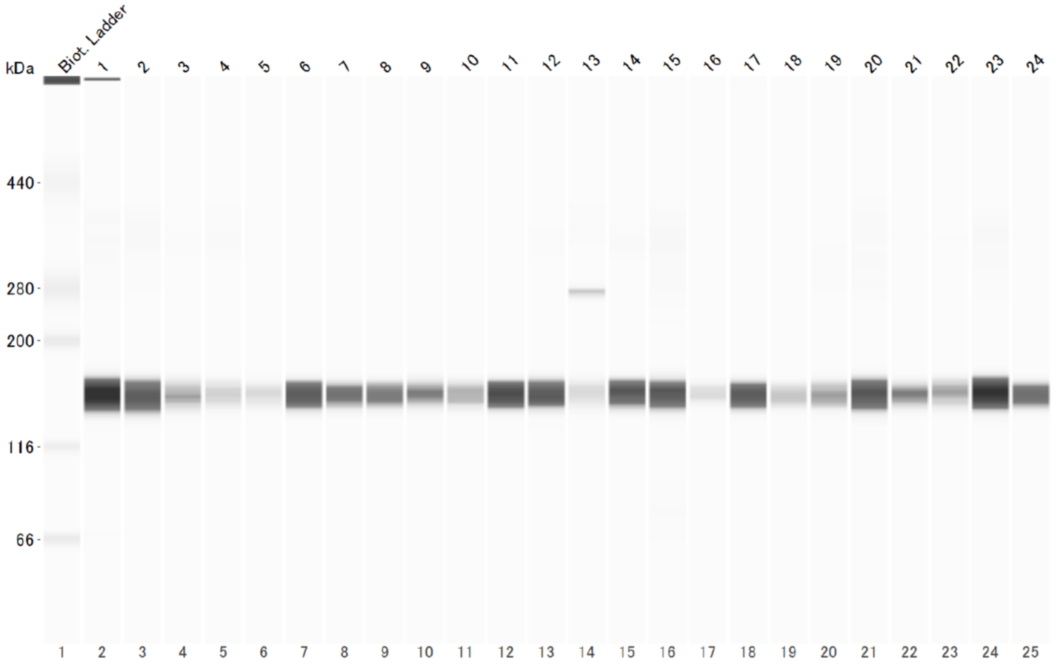

FANCA

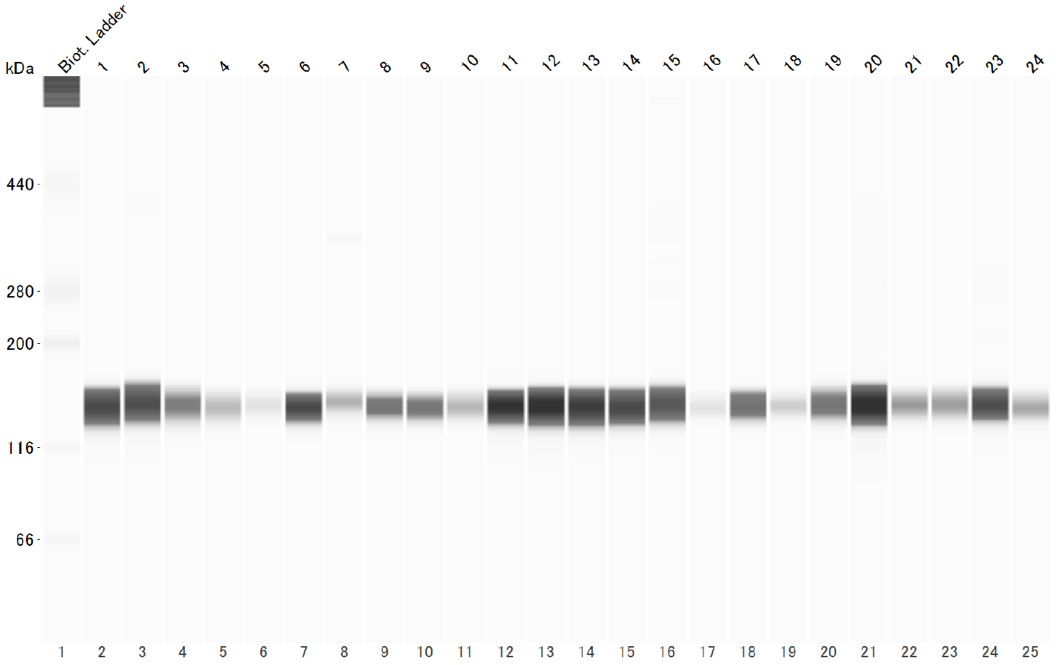

Supplemental Fig. 13 (continued)

γH2AX

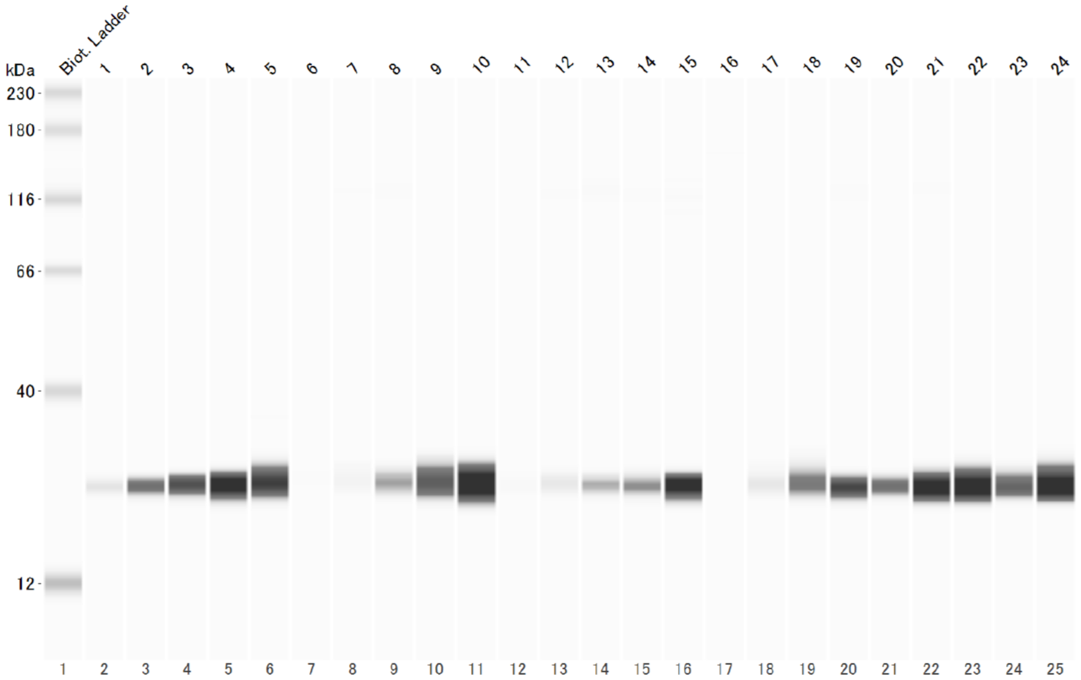

Cleaved
Caspase-3

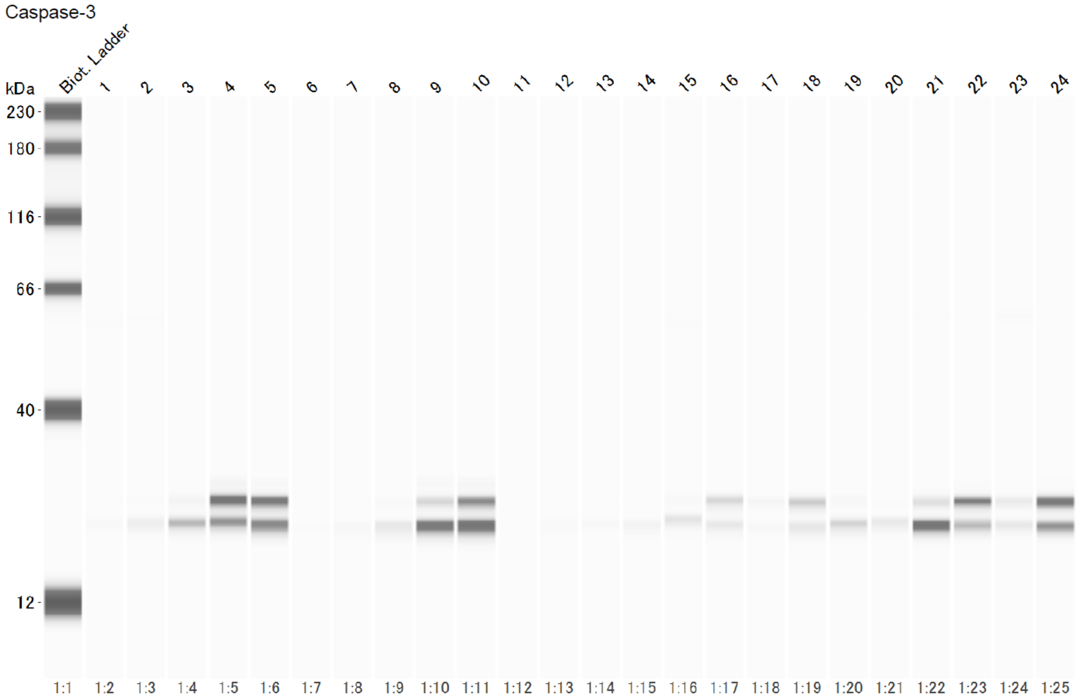

Supplemental Fig. 13 (continued)

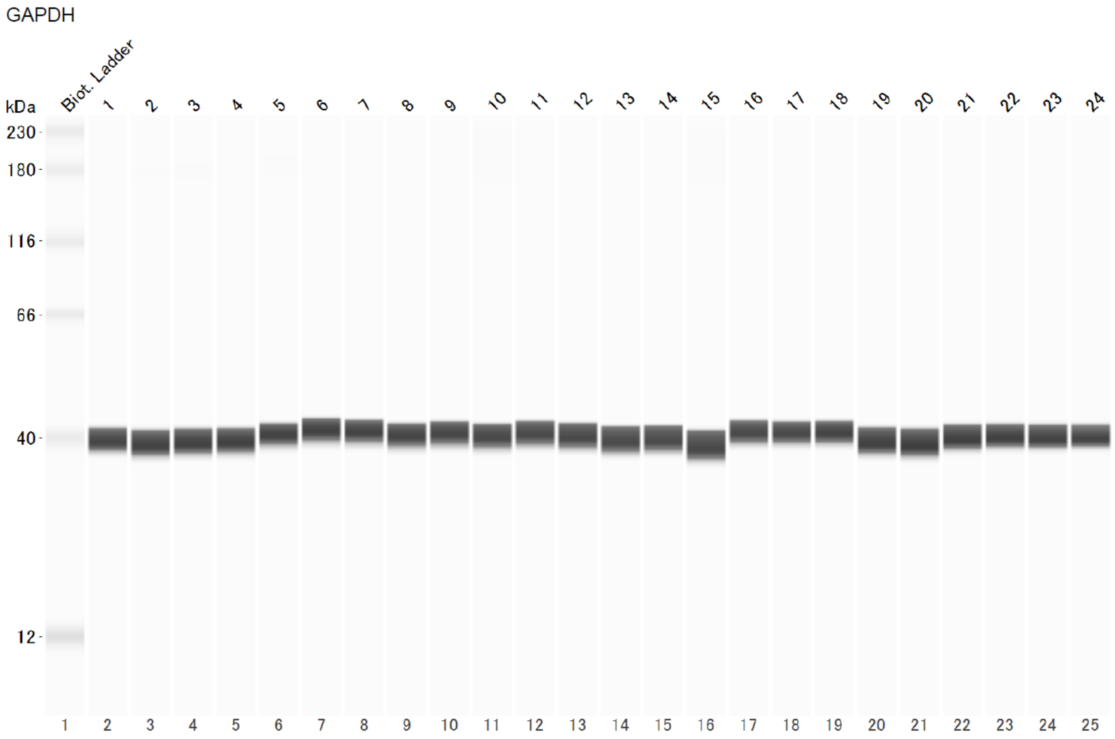

Lane	Cell	Time point	Drug	Figure
1	DLD1-P	0 hrs	E7820 1μM	N.A.
2	DLD1-P	24hrs	E7820 1μM	N.A.
3	DLD1-P	48hrs	E7820 1μM	N.A.
4	DLD1-P	72hrs	E7820 1μM	N.A.
5	DLD1-P	120hrs	E7820 1μM	N.A.
6	DLD1-KO	0 hrs	E7820 1μM	Fig. 3g
7	DLD1-KO	24hrs	E7820 1μM	Fig. 3g
8	DLD1-KO	48hrs	E7820 1μM	Fig. 3g
9	DLD1-KO	72hrs	E7820 1μM	Fig. 3g
10	DLD1-KO	120hrs	E7820 1μM	Fig. 3g
11	DLD1-KO-ER	0 hrs	E7820 1μM	N.A.
12	DLD1-KO-ER	24hrs	E7820 1μM	N.A.
13	DLD1-KO-ER	48hrs	E7820 1μM	N.A.
14	DLD1-KO-ER	72hrs	E7820 1μM	N.A.
15	DLD1-KO-ER	120hrs	E7820 1μM	N.A.
16	DLD1-P	72hrs	E7820 1μM	Fig. 4c
17	DLD1-P	72hrs	Olaparib 1μM	Fig. 4c
18	DLD1-P	72hrs	E7820 1μM + Olaparib 1μM	Fig. 4c
19	DLD1-KO	72hrs	E7820 1μM	Fig. 4c
20	DLD1-KO	72hrs	Olaparib 1μM	Fig. 4c
21	DLD1-KO	72hrs	E7820 1μM + Olaparib 1μM	Fig. 4c
22	DLD1-KO-OR	72hrs	E7820 1μM	Fig. 4c
23	DLD1-KO-OR	72hrs	Olaparib 1μM	Fig. 4c
24	DLD1-KO-OR	72hrs	E7820 1μM + Olaparib 1μM	Fig. 4c
