# Supplementary material for: A molecular glue RBM39-degrader induces synthetic lethality in cancer cells with homologous recombination repair deficiency
Source: NPJ Precis Oncol. 2024 May 24;8:117. doi: 10.1038/s41698-024-00610-0 (PMC11126574; doi:10.1038/s41698-024-00610-0)
Supplement: Supplementary file 7 — Reporting summary [file 41698_2024_610_MOESM7_ESM.pdf]

nature portfolio

Corresponding author(s): Shinji Kohsaka

Last updated by author(s): 2024/3/25

Reporting Summary

Nature Portfolio wishes to improve the reproducibility of the work that we publish. This form provides structure for consistency and transparency in reporting. For further information on Nature Portfolio policies, see our Editorial Policies and the Editorial Policy Checklist.

Statistics

For all statistical analyses, confirm that the following items are present in the figure legend, table legend, main text, or Methods section.

n/a	Confirmed	
☐	☒	The exact sample size (*n*) for each experimental group/condition, given as a discrete number and unit of measurement
☐	☒	A statement on whether measurements were taken from distinct samples or whether the same sample was measured repeatedly
☐	☒	The statistical test(s) used AND whether they are one- or two-sided<br>*Only common tests should be described solely by name; describe more complex techniques in the Methods section.*
☐	☒	A description of all covariates tested
☐	☒	A description of any assumptions or corrections, such as tests of normality and adjustment for multiple comparisons
☐	☒	A full description of the statistical parameters including central tendency (e.g. means) or other basic estimates (e.g. regression coefficient) AND variation (e.g. standard deviation) or associated estimates of uncertainty (e.g. confidence intervals)
☐	☒	For null hypothesis testing, the test statistic (e.g. *F*, *t*, *r*) with confidence intervals, effect sizes, degrees of freedom and *P* value noted<br>*Give P values as exact values whenever suitable.*
☒	☐	For Bayesian analysis, information on the choice of priors and Markov chain Monte Carlo settings
☐	☒	For hierarchical and complex designs, identification of the appropriate level for tests and full reporting of outcomes
☐	☒	Estimates of effect sizes (e.g. Cohen's *d*, Pearson's *r*), indicating how they were calculated

Our web collection on statistics for biologists contains articles on many of the points above.

Software and code

Policy information about availability of computer code

Data collection	No unreported custom computer code or algorithm was used to generate the results of this manuscript.
Data analysis	N/A

For manuscripts utilizing custom algorithms or software that are central to the research but not yet described in published literature, software must be made available to editors and reviewers. We strongly encourage code deposition in a community repository (e.g. GitHub). See the Nature Portfolio guidelines for submitting code & software for further information.

Data

Policy information about availability of data

All manuscripts must include a data availability statement. This statement should provide the following information, where applicable:

- Accession codes, unique identifiers, or web links for publicly available datasets
- A description of any restrictions on data availability
- For clinical datasets or third party data, please ensure that the statement adheres to our policy

We have deposited the raw sequencing data in the Japanese Genotype-Phenotype Archive (http://trace.ddbj.nig.ac.jp/jga), which is hosted by the DNA Data Bank of Japan.

Research involving human participants, their data, or biological material

Policy information about studies with human participants or human data. See also policy information about sex, gender (identity/presentation), and sexual orientation and race, ethnicity and racism.

Reporting on sex and gender	N/A
Reporting on race, ethnicity, or other socially relevant groupings	N/A
Population characteristics	N/A
Recruitment	N/A
Ethics oversight	N/A

Note that full information on the approval of the study protocol must also be provided in the manuscript.

Field-specific reporting

Please select the one below that is the best fit for your research. If you are not sure, read the appropriate sections before making your selection.

☒ Life sciences ☐ Behavioural & social sciences ☐ Ecological, evolutionary & environmental sciences

For a reference copy of the document with all sections, see nature.com/documents/nr-reporting-summary-flat.pdf

Life sciences study design

All studies must disclose on these points even when the disclosure is negative.

Sample size	Sample size was described in the manuscript.
Data exclusions	Data exclusions were properly described in the manuscript.
Replication	For next-generation sequencing, no replicate was performed.
Randomization	Randomization was not performed for this study.
Blinding	Blinding in this study was not performed. Data analysis were performed by the computational pipeline.

Reporting for specific materials, systems and methods

We require information from authors about some types of materials, experimental systems and methods used in many studies. Here, indicate whether each material, system or method listed is relevant to your study. If you are not sure if a list item applies to your research, read the appropriate section before selecting a response.

Materials & experimental systems

n/a	Involved in the study	
☐	☒	Antibodies
☐	☒	Eukaryotic cell lines
☒	☐	Palaeontology and archaeology
☒	☐	Animals and other organisms
☒	☐	Clinical data
☒	☐	Dual use research of concern
☒	☐	Plants

Methods

n/a	Involved in the study	
☒	☐	ChIP-seq
☒	☐	Flow cytometry
☒	☐	MRI-based neuroimaging

Antibodies

Antibodies used	Primary antibodies mouse anti-γH2AX (#613402, BioLegend, San Diego, CA, USA), rabbit anti-Rad51 (#ab133534, Abcam, Cambridge, UK), rabbit anti-phospho RPA2 (Ser33) (#NB100-544, Novus Biologicals, Littleton, CO, USA), or rabbit anti-53BP1 (#PC712, Merck) were used for immunofluorescence.RBM39 (HPA001591, atlas antibodies, Bromma, Sweden), phospho-histone H2A. X (Ser139)

	(20E3, #9718, Cell Signaling Technology), FANCA (D1L2Z, #14657, Cell Signaling Technology), FANCD2 (D5L5X, #16323, Cell Signaling Technology), and GAPDH (14C10, #2118, Cell Signaling Technology) were used for western blotting.
Validation	The antibody used in immunohistochemistry was obtained from commercial sources. Please see manufacturer's link for validation of antibody.

Eukaryotic cell lines

Policy information about cell lines and Sex and Gender in Research

Cell line source(s)	Human colorectal adenocarcinoma cell line DLD1 parental cells and homozygous BRCA2 (−/−) variant cells were purchased from Horizon Discovery, Inc (Cambridge, UK). PC9 was provided by the RIKEN BRC (Ibaraki, Japan) through the National BioResource Project of the MEXT, Japan. KKU-213 and KMLS1were provided from the JCRB Cell Bank. (Osaka, Japan). YaFuSS, derived from synovial sarcoma, was kindly provided by Dr J. Toguchida (Kyoto University, Japan). FUJI was a gift from Dr S. Tanaka (Hokkaido University, Japan). H2228, HCC1937 and HCC1428 were purchased from the American Type Culture Collection (Manassas, VA, USA).
Authentication	None of the cell lines used were authenticated within recent six months.
Mycoplasma contamination	Cell lines were not tested for mycoplasma contamination within recent six months.
Commonly misidentified lines (See ICLAC register)	N/A

Plants

Seed stocks	N/A
Novel plant genotypes	N/A
Authentication	N/A
